# Research Progress on Photosensitizers for DSSC

**DOI:** 10.3389/fchem.2018.00481

**Published:** 2018-10-11

**Authors:** Antonio Carella, Fabio Borbone, Roberto Centore

**Affiliations:** Chemical Sciences Department, University of Naples Federico II, Naples, Italy

**Keywords:** photovoltaics, dye sensitized solar cells, ruthenium polypyridyl complexes, Zn-porphyrin dyes, metal free organic dyes

## Abstract

Dye sensitized solar cells (DSSC) are considered one of the most promising photovoltaic technologies as an alternative to traditional silicon-based solar cells, for their compatibility with low-cost production methods, their peculiar optical and mechanical properties and the high indoor efficiency. Photosensitizers represent one of the most important components of a DSSC device and probably the most thoroughly investigated in the last twenty years, with thousands of dyes that have been proposed and tested for this kind of application. In this review we aimed to provide an overview of the three main classes of DSSC photosensitizers, namely ruthenium(II) polypyridyl complexes, Zn-porphyrin derivatives and metal-free organic dyes. After a brief introduction about the architecture and operational principles of a DSSC and the state of the art of the other main components of this type of device, we focused our discussion on photosensitizers. We have defined the numerous requirements DSSC photosensitizers should satisfy and have provided an overview of their historical development over the years; by examining specific dyes reported in the literature, we attempted to highlight the molecular design strategies that have been established for the optimization of their performance in real devices both in terms of efficiency (which recently reaches an outstanding 14.3%) and operational stability. Finally, we discussed, in the last section, the possible future developments of this intriguing technology.

## Introduction

One of the major societal challenges of the coming years is related to the rise in global energy demands: the latest International Energy Outlook 2017 of U.S. Energy Information Administration (available from: https://www.eia.gov/outlooks/ieo) projects that the world energy consumption will grow by 28% between 2015 and 2040, going from 19.2 to 24.6 TWy. The fast depletion of fossil fuels reserves, together with the severe environmental impact associated with their combustion, push the development of energy production technologies based on clean and renewable energy sources. In this context solar energy appears to be the most obvious answer: the sun provides 120000 TWy of energy each year to the Earth's surface (Lewis et al., 2005), which means that all the energy man activities require in 1 year is obtained in less than 1.5 h. There is no other energy source as abundant as solar energy; moreover, it can be considered inexhaustible on the scale of humankind and received at no cost. These are the reasons why photovoltaics (PV) represents one the fastest growing markets: the compound annual growth rate of PV plants was 40% in the period 2010–2016, according to Fraunhofer PV Report 2017 (https://www.ise.fraunhofer.de/content/dam/ise/de/documents/publications/studies/Photovoltaics-Report.pdf).

Silicon based solar cells today dominate the PV market, accounting for the 93% of the total PV plants: PV devices based on highly crystalline silicon have achieved an efficiency of 26.7%, (Green et al., 2018) which is close to the maximum theoretical efficiency limit (31%) determined by Shockley and Queisser (1961). However, the silicon required for solar cells must be extremely pure and is obtained through expensive high temperature and high vacuum processes.

These constraints have pushed the development of alternative PV technologies based on low cost processing and materials. The Dye Sensitized Solar Cells (DSSC) today represent one of the most promising PV technologies alternative to traditional silicon based solar cells. DSSC present several elements of interest: devices can be fabricated using procedures based on low cost solutions, such as inkjet or screen printing (Hashmi et al., 2015, 2016; Mariani et al., 2015), compatible with conventional roll-to-roll techniques (Hashmi et al., 2011). With such techniques large area devices can be prepared on flexible substrates (Chai et al., 2015; Li et al., 2016), even on textiles (Yun et al., 2015) and paper (Wang and Kerr, 2011), thus opening the way to applications in the field of wearable/portable electronics. The DSSC devices are also semitransparent and can be made in a range of different colors. These features, together with the flexibility mentioned above, make them compatible with architectural elements different from the roof and therefore extremely attractive in the field of building integrated photovoltaics (Saifullah et al., 2016). A further strength of DSSC lies in their extremely high performance in indoor conditions under artificial light compared to other PV technologies: an indoor efficiency up to 28.9% has been achieved (under 1000 lux indoor illumination) which could provide sufficient power to allow the autonomous operation of small electronic devices (Freitag et al., 2017).

The seminal work that has driven the development of DSSC was presented in 1991 by O'Regan and Grätzel (1991). The photo-electrochemical cell described by the authors was based on the sensitization of a wide bandgap metal oxide semiconductor, TiO_2_, with a ruthenium based metallo-organic dye. The main breakthrough introduced in this paper was the use of a mesoporous film of TiO_2_ nanoparticles that lead to a significant increase in efficiency up to 7%: the increased surface area of the semiconductor (compared to what previously reported in this field) allowed the adsorption of a greater amount of dyes and was the main reason for this change in efficiency. Since then an impressive number of papers have been reported in the literature that have demonstrated efforts to obtain more efficient DSSCs by properly intervening on the different components of this kind of device (metal oxide semiconductor, sensitizers, electrolytes). In this review we will focus in particular on the development of sensitizers and their rational design rules behind the achievement of more efficient and long term stable devices.

## Dye sensitized solar cells: working principles and materials

### Architecture and operation principles

**Figure 1:**
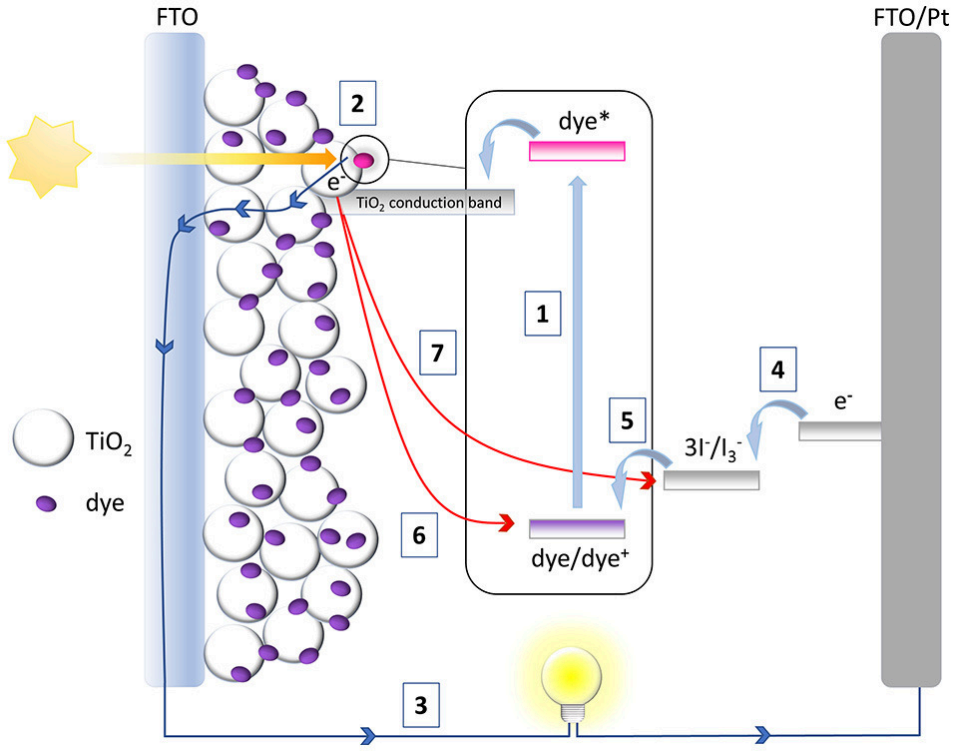
Architecture and operation principles of a typical DSSC.

The architecture of a classical DSSC is shown in Figure 1. The heart of the device is a thin layer of a mesoporous metal oxide semiconductor sensitized with a metallo-organic or organic dye. This mesoporous layer is deposited on the top of a transparent electrode which in turn is supported on a suitable substrate (glass, plastic, etc.). This assembly constitutes the working electrode of the device, the photoanode. The counter-electrode of the device consists of a transparent conductive layer deposited on a suitable substrate. An electrolyte solution containing a redox mediator, is sandwiched between the two electrodes and, by filling the porous structure of the semiconductor, allows the electrical contact between them. The operation of a DSSC is based on a multistep process as outlined in Figure 1. The first step (1) involves the absorption of a photon by the sensitizer that determines the promotion of an electron in the excited state. An electron is then injected from the excited sensitizer into the conduction band of the semiconductor (2), thus leaving the sensitizer in the oxidized state. The injected electron percolates through the mesoporous structure of the semiconductor, driven by a chemical diffusion gradient (Hagfeldt et al., 2010) and it is collected at the transparent conductive electrode and then transferred to the external circuit (3). Through the external circuit the electron reaches the counter-electrode and here interacts with the redox mediator turning it in its reduced form (4). The reduced state of the redox mediator finally reduces the oxidized sensitizer, regenerating the original dye and completing the circuit (5).

The DSSC is hence a conservative photo-electrochemical cell that produces electrical current without changing the chemical composition of the device. Some undesirable processes can anyway occur during DSSC operation: in particular, the charge recombination of the injected electrons with the oxidized sensitizer (6) or with the oxidized state of the redox couple (7, the consequence of which is the so called “dark current”) makes the absorbed photon (responsible for the injected electron) useless for the production of electrical work, thus reducing device performance. The kinetic competition between the favorable and unwanted processes described above ultimately determines the overall efficiency of the device. The cell efficiency is described as the ratio between the maximum output power of the device and the intensity of the incident light (I_S_): it is related to some electrical parameters that can be extracted by a classical current intensity vs. voltage (*IV*) experiment performed under simulated solar radiation, as indicated in Equation (1):

(1) $$\begin{array}{r} \eta =J_{SC}\times V_{OC}\times FF/I_{s} \end{array}$$

*J*_*SC*_ is the photocurrent density determined in short circuit conditions; it is related to the electron injection yield that, in turn, depends on the absorption properties of the sensitizer (qualitatively and quantitatively) and on a favorable alignment of the sensitizer LUMO level with the semiconductor conduction. *V*_*OC*_ represents the open circuit voltage and depends on the energy difference between the Fermi potential (under illumination) of the semiconductor and the Nernst potential of the redox couple. The fill factor *FF* is expressed as the ratio between the maximum power *P*_*MAX*_ deliverable by the device and the maximum theoretical power (*P*_*TH*_ = *J*_*SC*_ × *V*_*OC*_) and reflects the electrical and electrochemical losses that occur during device operation.

### The components of a DSSC device

A DSSC can be viewed as a multicomponent device and all the components have been intensively investigated, both individually and as regarding the interaction with the other components, in order to better understand the operation principles and improve the efficiency of the cell. This review will discuss in detail the role and the development of sensitizers. A brief preliminary discussion on the state of the art of the other components of the device will however be in the following lines.

#### The photoanode

As for the working electrode of a classic DSSC, it can be considered formed by the combination of three different materials: a transparent conductive electrode (TCE), a mesoporous semiconductor layer and the adsorbed dye. Typically used TCEs are based on micrometric films of doped metal oxide semiconductors, supported on glass or flexible substrates. In particular, fluorine doped SnO_2_, FTO, is the most used electrode for DSSC because of its high thermal resistance, electrical conductivity and optical transparency (Muthukumar et al., 2013). More recently, alternative materials to FTO have been explored, such as graphene based electrodes, which can ensure better compatibility with flexible substrates (Roy-Mayhew and Aksay, 2014; Guo et al., 2015). The second component of the working electrode, a mesoporous n-type semiconductor, is deposited on the top of the TCE. The typical semiconductor metal oxide used is TiO_2_, in its anatase crystalline form, which is the same employed in the pioneering work of O'Regan and Grätzel (1991). Interestingly, despite a huge research effort in the search for alternative semiconductor materials such as ZnO (Anta et al., 2012; Canto-Aguilar et al., 2017; Vittal and Ho, 2017) and SnO_2_ (Pari et al., 2013; Li et al., 2014), TiO_2_ still remains the material that provides the best efficiency in photovoltaic devices (Bai et al., 2014; Cavallo et al., 2017). It is also an abundant material characterized by well-established low-cost synthetic preparation procedures. A wide range of TiO_2_ nanostructures was investigated to improve charge mobility including nanotubes (Roy et al., 2010), nanorods (Lv et al., 2013), or nano-opals (Ha et al., 2014). The best performing DSSC uses a double layer mesoporous TiO_2_ film: a light-absorbing layer composed of 15–20 nm sized anatase particles and a light-scattering over-layer composed of 200–400 nm sized anatase particles (Bai et al., 2014) which have a beneficial effect on the overall light-harvesting. The third component of the working electrode consists of the sensitizers that will be described in more detail in the following paragraphs.

#### Electrolyte

The electrolyte solution is responsible for dye regeneration and hole transport during DSSC device operation. Liquid electrolytes are the most investigated systems in DSSC field. They are basically composed of three main elements: a solvent, a redox mediator (ionic conductor) and various additives (Wu et al., 2015). The electrolyte solvent must meet certain requirements such as melting point below −20°C and oiling point above 100°C (to reduce loss by means of evaporation), chemical and photochemical stability, high dielectric constant to allow solubilization of the electrolytic salts and low viscosity to promote high diffusion coefficients of the redox mediators. Several organic solvents have been used for the electrolyte solution including ethanol, acetonitrile, organic nitriles and carbonates (Yu et al., 2011). Acetonitrile (AN) is considered the best solvent for fundamental studies when maximizing cell efficiency is sought (Hagfeldt et al., 2010). At the same time AN is not ideal for the study of the stability of the device, because of its low boiling point (82°C). In this case, a higher boiling point organic nitrile such as methoxypropionitrile (MPN) is widely used. Solvents based on ionic liquids and particularly on imidazolium salts have been widely studied as well: they possess a high chemical and electrochemical stability, an excellent ionic conductivity and a very low vapor pressure which reduces solvent evaporation and leakage. As a consequence of higher viscosity, however, they provide lower efficiency than organic solvents and a mixture of the two classes of solvents is often used (Gorlov and Kloo, 2008).

As for the redox couple, it should possess adequate thermodynamic characteristics to allow an efficient dye regeneration and it determines the V_oc_ value, together with the semiconductor used. Historically, the most commonly used redox mediator is iodide/triiodide couple (I^−^/I${}_{3}^{-}$) which is characterized by a very slow recombination kinetic with the electron injected into the TiO_2_ conduction band (Boschloo and Hagfeldt, 2009). However, the use of different redox mediators has been widely investigated: in particular cobalt based (Co^II^/Co^III^) complexes represent the redox mediator providing DSSC devices with higher efficiencies (Giribabu et al., 2015; Bella et al., 2016), which is mainly related to an increased Nernst potential and a theoretical higher achievable V_OC_.

The electrical additives represent another important component of liquid electrolytes: they act by modulating some characteristics of the system such as the edge of the conduction band (CB) of the semiconductor, the recombination kinetics, the photovoltaic parameters. In particular, the use of nitrogen-containing heterocycles causes an upward shift of TiO_2_ CB, with a consequent increase in V_OC_ (Boschloo et al., 2006). At the same time the use of additives containing specific cations (such as Li^+^ or guanidinium) helps to improve the electron injection rate by shifting TiO_2_ CB toward lower energies, thus increasing the driving force for the process of electron injection and thus J_SC_ (Boschloo et al., 2006).

The use of liquid electrolytes poses some practical problems due to solvent leakage or evaporation which could negatively affect long term stability of the device; the use of quasi solid or solid state electrolyte could be the answer to these problems and have been thoroughly investigated in the last years (Wu et al., 2015). Quasi solid-state electrolytes are typically prepared by “solidifying” organic solvents (or ionic liquids) in a polymer or oligomer that acts as gelator. They show better long-term stability than liquid electrolytes, preserving the high ionic conductivity and interfacial contact properties with the semiconductor (Wang, 2009). The solid state DSSC are instead based on a hole transporter, inorganic or organic material (HTM), which replaces the liquid electrolyte: here, the hole transfer occurs directly between the oxidized dye and the HTM and the holes are transferred toward the cathode through the HTM. The efficiency of this kind of devices is still lower than the liquid-electrolyte based DSSC mainly due to an inefficient infiltration of the HTM into the semiconductor pores, which reduces the optimal thickness of TiO_2_ film to only 2 μm (Snaith and Schmidt-Mende, 2007): therefore, dyes with a very high molar extinction coefficients are required.

#### The counter electrode (cathode)

The main function of the cathode is to provide electrons from the external circuit to reduce the oxidized form of the redox couple. Typically, the counter electrode is typically composed of a micrometric layer of FTO deposited on a mechanical support.

To obtain sufficiently fast kinetics for the reduction of the redox couple, a catalyst coating is necessary: to date, platinum is the best material of choice (Wu et al., 2017). Pt can be deposed by several methods as electrodeposition, spray pyrolysis, sputtering, and vapor deposition (Hagfeldt et al., 2010). Superior performances have been achieved with nanoscale Pt clusters prepared by thermal decomposition of platinum chloride compounds With a cost of about 25,000 $ per kilo, platinum represents the most expansive element used in the fabrication of DSSC. In view of a desirable large-scale production of DSSC, different and cheaper catalysts need to be optimized. In this context nanocarbon materials as graphene (Wang and Hu, 2012; Tasis, 2017) and carbon nanotubes (Hwang et al., 2015; Al-Marzouki et al., 2016) have been investigated with promising results.

## Sensitizers

Sensitizers represent one of the most important elements of a DSSC. The main role of the sensitizers is the absorption of solar photons and the injection of the photoexcited electrons into the conduction band of the n-type semiconductor. Dye sensitizers should fulfill several requirements to efficiently perform in a DSSC:

- Their optical absorption should cover a large part of the visible spectrum and extend up to the NIR region; high molar extinction coefficients are desired to achieve efficient solar photons harvesting.
- Their molecular structure should be characterized by peripheral anchoring groups, typically of acidic nature, which allow a firm adhesion to the semiconductor surface.
- The excited state level of the dye sensitizer should be higher in energy than the conduction band edge of n-type semiconductor so that an efficient electron injection process from the excited dye into the conduction band of the semiconductor can occur.
- The HOMO of the photosensitizer should lie below the energy level of the redox mediator to promote dye regeneration.
- The photosensitizers should feature high photostability to resist the continuous light soaking; thermal and electrochemical stability are also required.

A great variety of photosensitizers for DSSC applications have been studied in the last decades: they are probably the most investigated component of a DSSC device and have been excellently reviewed in several papers (Mishra et al., 2009; Albero et al., 2015; Higashino and Imahori, 2015). In this review we will focus on the three main classes of DSSC photosensitizers, Ru(II) polypyridyl complexes, Zn porphyrin derivative and metal-free organic dyes, describing their development and highlighting the design rules that have allowed the obtainment of highly performant and stable molecular systems.

### Ruthenium based photosensitizers

**Figure 2:**
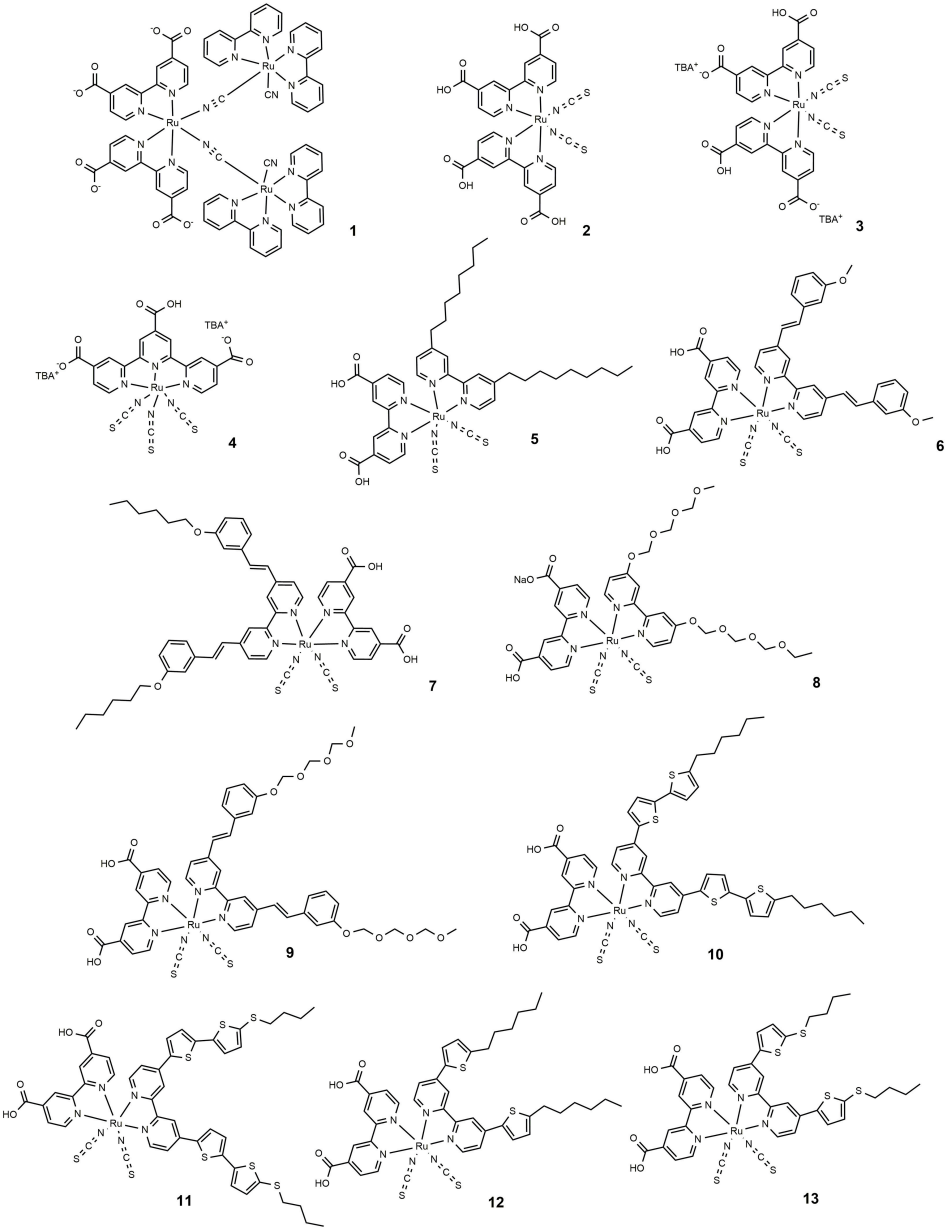
Chemical structure of dyes 1–13.

Among the DSSC organometallic photosensitizers, the Ru(II) polypyridyl complexes are by far the most studied: their peculiar photophysical and redox properties are the reasons of this intense investigation (Reynal and Palomares, 2011; Qin and Peng, 2012). Specifically, their excellent photovoltaic behavior derives from a broad absorption spectrum, ideally aligned excited and ground states and stability in the oxidized state (Hagfeldt et al., 2010). Their absorption properties, related to a metal to ligand charge transfer (MLCT) process can be engineered by suitable modification of the ligands so that a large part of the visible spectrum can be covered. Ru complexes with carboxylate ligands have been studied as sensitizers of wide bandgap semiconductor since 1979 (Anderson et al., 1979; Desilvestro et al., 1985). In 1991 O'Regan and Grätzel employed a previously synthesized (Amadelli et al., 1990; Nazeeruddin et al., 1990) Ru trinuclear complex with improved absorption properties (dye **1** in Figure 2) in the manufacture of the first efficient DSSC (O'Regan and Grätzel, 1991; O'Regan et al., 2009).

In 1993 Nazeeruddin et al. report a very important study on the optical and electrochemical properties of a series of Ru (II) complexes of general formula *cis*-X_2_bis(2,2′-bipyridyl-4,4′-dicarboxylate)-Ru(II) where X = Cl, Br, I, CN, and SCN (Nazeeruddin et al., 1993). One of these, the one containing two groups of isocyanate ligands (dye **2**, Figure 2) showed exceptional photovoltaics properties: the use of two thiocyanate groups instead of other ligands produced a red shift of the dye absorption spectrum up to 800 nm: this extended absorption was at the origin of a very high J_SC_ of 18 mA/cm^2^ and an overall efficiency of 10% (as reported in Table 1).

**Table 1:** Photovoltaics parameters of DSSC sensitized Ru-based sensitizers dyes 1–20 and 36.

**Dye**	**Code**	**J_SC_**	**V_OC_**	**FF**	**η**	**λ_MAX_(nm)/ε × 10^−3^**	**References**
		**(mA·cm^−2^)**	**(V)**		**(%)**	**(M^−1^cm^−1^)**	
1	–	–	–	0.685	7.12	–	O'Regan and Grätzel, 1991
2	N3	18.2	0.72	0.73	10.0	313/31.2, 396/14.0, 534/14.2	Nazeeruddin et al., 1993
3	N719	17.73	0.846	0.75	11.2	312/49.1, 380/1.33, 538/1.42	Buscaino et al., 2008
4	N749	20.53	0.72	0.704	10.4	411/–, 536/–, 610/7.48	Nazeeruddin et al., 2001
5	Z907	14.2	0.764	0.676	7.8	312/25.0, 384/10.1, 525/11.1^a^	Wang et al., 2003a
6	Z910	17.2	0.777	0.764	10.2	410/17.01, 543/16.85	Wang et al., 2004
7	K19	14.61	0.711	0.671	7.0	543/18.2	Wang et al., 2005a
8	K51	16.6	0.738	0.679	8.1	–	Kuang et al., 2006
9	K60	16.7	0.715	0.69	8.44	310/–, 356/–, 547/18.55	Kuang et al., 2007
10	CYC-B1	23.92	0.65	0.54	8.54	312/35.8, 400/46.4, 553/21.2	Chen et al., 2006
11	CYC-B11	20.05	0.743	0.77	11.5	305/–, 388/–, 554/24.2	Chen et al., 2009
12	C101	10.5	0.747	0.76	11.7	305/–, 341/–, 407/–, 547/17.5^b^	Sauvage et al., 2011
13	C106	18.28	0.749	0.772	10.57	310/–, 348/–, 550/18.7	Cao et al., 2009
14	RC43	20.21	0.725	0.73	10.78	418/64.3, 557/27.4	Chen et al., 2016
15	Z1	17.7	0.74	0.66	10.2	301/50.8, 356/40.8, 525/13.8	Lu et al., 2016b
16	SCZ1	19.88	0.761	0.688	10.4	306/64.9, 407/30.6, 539/17.7	She et al., 2015
17	–	17.01	0.800	0.740	10.1	406/–, 490/–, 560/16.7	Bessho et al., 2009
18	–	16.74	0.682	0.710	8.0	–	Robson et al., 2011
19	TFRS2	17.15	0.82	0.678	9.54	424/23.4, 460/21.9, 533/16.4	Wu et al., 2010
20	D-CF3	19.50	0.704	0.65	8.74	406/–, 500/–, 562/12.0	Huang et al., 2015
36	SA-246	14.55	0.845	0.747	9.4	382/36, 431/26.4, 585/21.5	Aghazada et al., 2016

a *Optical properties from Zakeeruddin et al. (2002)*.

b *Optical properties from Gao et al. (2008)*.

The effect of the proton content in dye **2** on the device efficiency was further investigated in 1999 (Nazeeruddin et al., 1999). It was found that the efficiency of the phosensitizers depends on its degree of protonation which influenced two important electrical parameters (J_SC_ and V_OC_) of the device, in opposite directions: after absorption of the dye, the surface of TiO_2_ is protonated and then positively charged. The surface dipole generated in this way favors the adsorption of the deprotonated anionic ruthenium complex and helps electron injection from the excited state of the sensitizer into the titania conduction band, determining high photocurrents. At the same time, the protonation of the TiO_2_ surface causes a positive shift of the conduction band edge which, in turn, lowers the V_oc_ of the device. The balance between these two factors led the double protonated derivative, **3**, to be the best performing photosensitizer in that paper and to offer, in a following work, a DSSC with an efficiency of 11.2% (Buscaino et al., 2008). Compounds **2** and **3** are still considered as references dyes in the DSSC field and have been used as a starting point for designing new Ru based photosensitizers by means of a suitable modification of ancillary ligands.

With the aim of further extending sensitizer absorption toward NIR, a new type of trithiocyanate-Ru(II) terpyridyl complexes was reported in 1997 (Nazeeruddin et al., 1997). The use of a further thiocyanate group compared to **2** originated dyes characterized by a panchromatic absorption covering the whole visible range and extending in the NIR region up to 920 nm (black dyes).

A derivative of this dye, double de-protonated, referred to as **N749** (dye **4** in Figure 2), has been efficiently employed as DSSC photosensitizer affording a device with an impressive J_SC_ of 20.5 mA/cm^2^ and an overall efficiency of 10.4% (Nazeeruddin et al., 2001).

The design of novel Ru based photosensitizers carrying hydrophobic groups in the ancillary ligands was explored to increase the stability of the dyes in the operation of the device. In 2003 the heteroleptic amphiphilic ruthenium complex **5** (Figure 2) was used in a DSSC device with good efficiency (about 8%) and remarkable long-term stability at 55°C under one sun illumination soaking (Wang et al., 2003a). The high stability of the device, in which a co-adsorbent (hexadecylmalonic acid) was grafted onto TiO_2_ together with the sensitizers, was due to the formation of a hydrophobic layer which hinders the desorption process of the dye by residual water and at the same time creates an insulating barrier between the semiconductor and the electrolyte, thus reducing the dark current. Dye **5** was also used in a quasi-solid state DSSC based on a polymer gel electrolyte, affording a device with 6% efficiency and an impressive stability: the cell was able to maintain 94% of its initial performance after 1,000 h at 80°C and 95% after 1,000 h under one sun light-soaking at 55°C (Wang et al., 2003b).

The use of hydrophobic tails with extended π-conjugation that replaced the alkyl tails was then explored to enhance the molar absorption of the dyes and thus the overall efficiency of the cell. One of these sensitizers was dye **6** (Figure 2) in which the bipyridyl ligand is functionalized with a methoxystyryl group (Wang et al., 2004). A red shift of 20 nm was obtained compared to **5**, and a higher molar extinction coefficient (even compared to **3**) which eventually led to a J_SC_ of 17.2 mA/cm^2^ and an overall efficiency of 10.2% (Table 1). A further evolution of structure **6** is represented by photosensitizer **7**, in which an hexyloxy tail replaced the methoxy group. The **7**-sensitized DSSC gave an efficiency of 7% with very high stability even in a liquid electrolyte (Wang et al., 2005a).

The introduction of ion coordinating tails was explored by Kuang et al. (2006, 2007, 2008). The molecular structure of dye **8** contains a triethylene oxide methyl ether group which has the ability to coordinate Li ions (typically used as additive in electrolyte solution) and inhibit them to reach the TiO_2_ surface which would result in a drop of photovoltage V_OC_. The efficiency of DSSC based on dye **8** was increased up to 8.1% by optimizing the concentration of Li^+^ in the electrolyte (Kuang et al., 2006). An increase in efficiency to 8.4% was achieved by extending the conjugation of the peripheral ligands in dye **9**. The device is also characterized by an excellent long-term stability when subjected to thermal stress at 80°C or one sun continuous illumination at 60°C (Kuang et al., 2007).

The use of thiophene groups in ancillary ligands have also been studied. The functionalization of the typical bipyridine ligands with a bithiophene moiety led to the sensitizer **10** which was used as a photosensitizer in a DSSC cell with an efficiency of 8.54% (Chen et al., 2006). The electron rich nature of thiophene heterocycle together with its high polarizability contributes to the good performance of the dye. The replacement of the alkyl chain of **10** with an electron rich thioalkyl group led to an even better photosensitizer, **11**, which afforded a device with 11.5% efficiency (Chen et al., 2009). The superior performance of **11** was mainly due to an improved molar extinction coefficient (2.42·10^4^ M^−1^ cm^−1^ in DMF solution): the increased absorptivity allowed the use of a TiO_2_ layer of reduced thickness which caused a decreased dark current leakage and an increase of V_OC_. The device based on **11** showed an excellent time stability under one sun at 60°C. The dye was also used in the assembly of a solid state DSSC (with a TiO_2_ layer of only 2 μm) characterized by an impressive efficiency of 4.7% (Chen et al., 2009). Other interesting examples of Ru-based photosensitizers containing thiophene are the dye **12** (Gao et al., 2008) and **13** (Cao et al., 2009) in which the bipyridine ligand is functionalized by a single thiophene ring: they are characterized by a high molar extinction coefficient (1.75·10^4^ M^−1^ cm^−1^ and 1.87·10^4^ M^−1^ cm^−1^ respectively for **12** and **13**). The efficiency of the device sensitized with **12** and **13** reached, respectively 11.0 and 11.4% in the cited papers. Interestingly, a further study on DSSC sensitized with **12** showed that the temperature of the sensitization process influenced the final efficiency of the device: an efficiency of 11.5% was obtained when **12** was adsorbed on TiO_2_ anode at 4°C (Cao et al., 2009). The sensitization process carried out at low temperature determined an increased structural order of the self-assembled dye layer which prevents the electron back transfer from TiO_2_ to triiodide ions (reducing the dark current).

**Figure 3:**
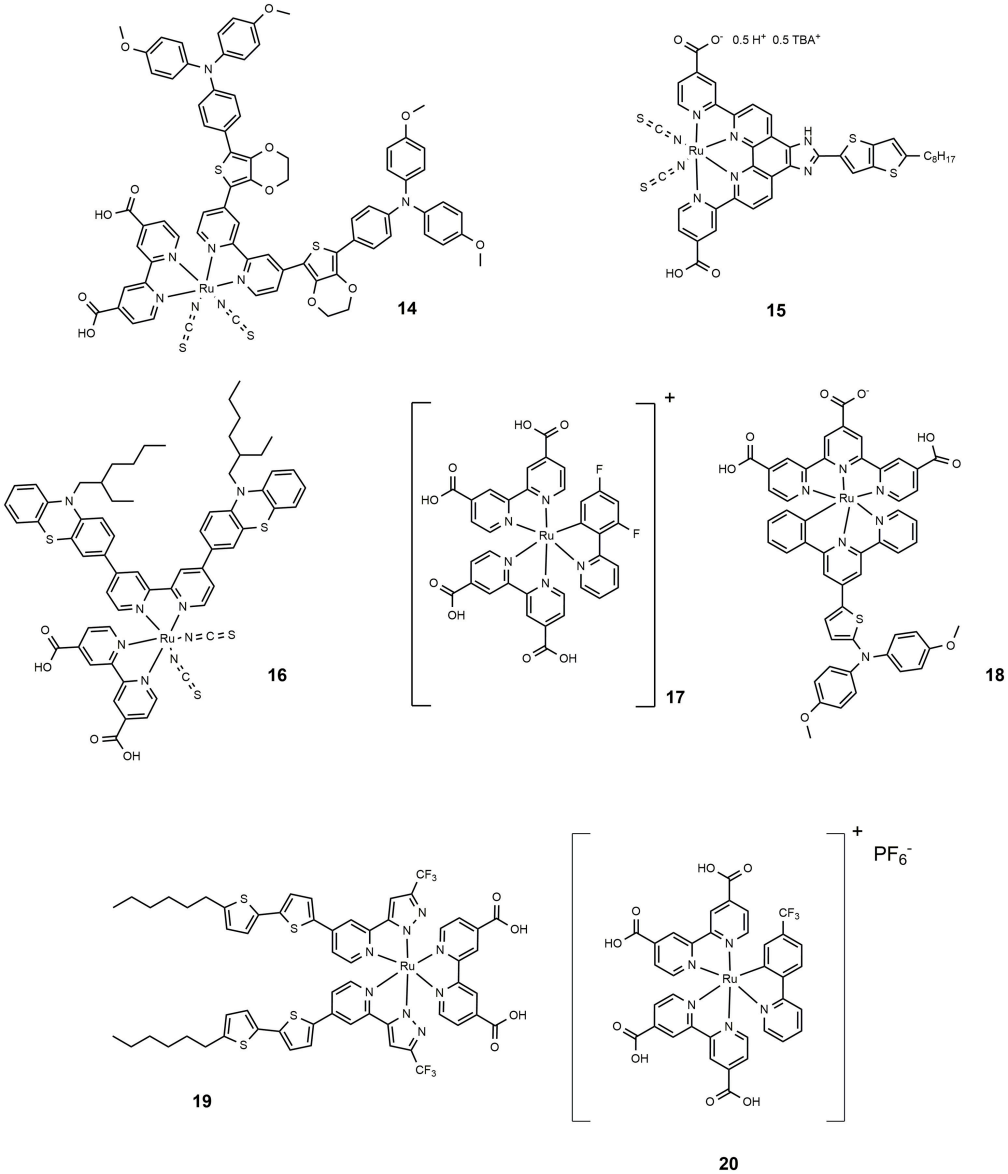
Chemical structure of dyes 14–20.

The incorporation of an electron delocalized donor antenna or hole transport chromophore in the ancillary ligands of a ruthenium polypyridyl complex sensitizer has proved an efficient route to increase the light-harvesting efficiency in MLCT band and red-shifting the absorption spectrum of ruthenium sensitizer. The molecular structure of **14** (shown in Figure 3) is characterized by a peripheral ring of ethylendioxythiophene (EDOT) functionalized with an electron donor moiety (triphenylamine like). This dye featured a molar absorption coefficient of 2.7·10^4^ M^−1^ cm^−1^ and showed excellent photovoltaic properties with an efficiency approaching 11% (Chen et al., 2016). Following this approach, other electron rich and polarizable heteroaromatics as thienothiophene (Lu et al., 2016b) or phenothiazine (She et al., 2015) have been tested as functionalising groups of ancillary ligands. Sensitizer **15** (Figure 3) afforded devices with 10.1% efficiency while **16** provided 10.4% efficiency.

The preparation of thiocyanate-free Ru-based sensitizers has also been investigated: the thiocyanate groups are considered the most fragile part of the ruthenium dyes typically used in DSSC (Nazeeruddin et al., 2011). The thiocyanate coordinates in fact through one atom and can be replaced by other competing ligands that produce less efficient species. Furthermore, it can coordinate both through sulfur and nitrogen atoms, making the purification steps of dyes more complicated. An example of high performance thiocyanate-free Ru sensitizers was reported in 2009: replacing the thiocyanate group with a cyclometalated 2,4-difluorophenylpyridine gave dye **17** (Figure 3), a panchromatic and robust dye that gave rise to a device with 10.1% efficiency (Bessho et al., 2009). The study, performed through electrochemical and spectroscopic analysis, of the effects of replacing a single polypyridyl ligand with an analogous cyclometalating anionic ligand, revealed that a bathochromic shift occurred following the destabilization of the dye HOMO which in turn is related to the strong electron donor ability of the cyclometalating ligand (Bomben et al., 2009). Furthermore, the cyclometalated complexes are particularly sensitive to *para* substitution at the central ring bearing the Ru-C bond (Koivisto et al., 2009). The study of a class of asymmetrical bistridentate-cyclometalated Ru(II) complexes bearing terminal triphenylamine substituents was reported in 2011 (Robson et al., 2011). Sensitization of devices with this class of complexes provided an efficiency of up to 8%, obtained using dye **18** (see Figure 3). Another interesting class of thiocyanate-free Ru-based photosensitizers was reported in 2012 (Wu et al., 2010): they are based on the replacement of bipyridine ligands with 2-pyridyl-pyrazolate ancillaries and exhibit light absorption properties up to 700 nm. The maximum power conversion efficiency measured for these systems is 9.5% when dye **19** (Figure 3) has been used as photosensitizer. Recently Huang et al. proposed a series of cyclometallated thiocyanate-free Ru photosensitizers based on phenylpyridine ligands and studied the effect of various substituents on the phenyl ring (Huang et al., 2015): the efficiency was found to depend on the electron acceptor ability of the substituent group and approached 9% for dye **20**. (Figure 3).

### Porphyrins based sensitizers

**Figure 4:**
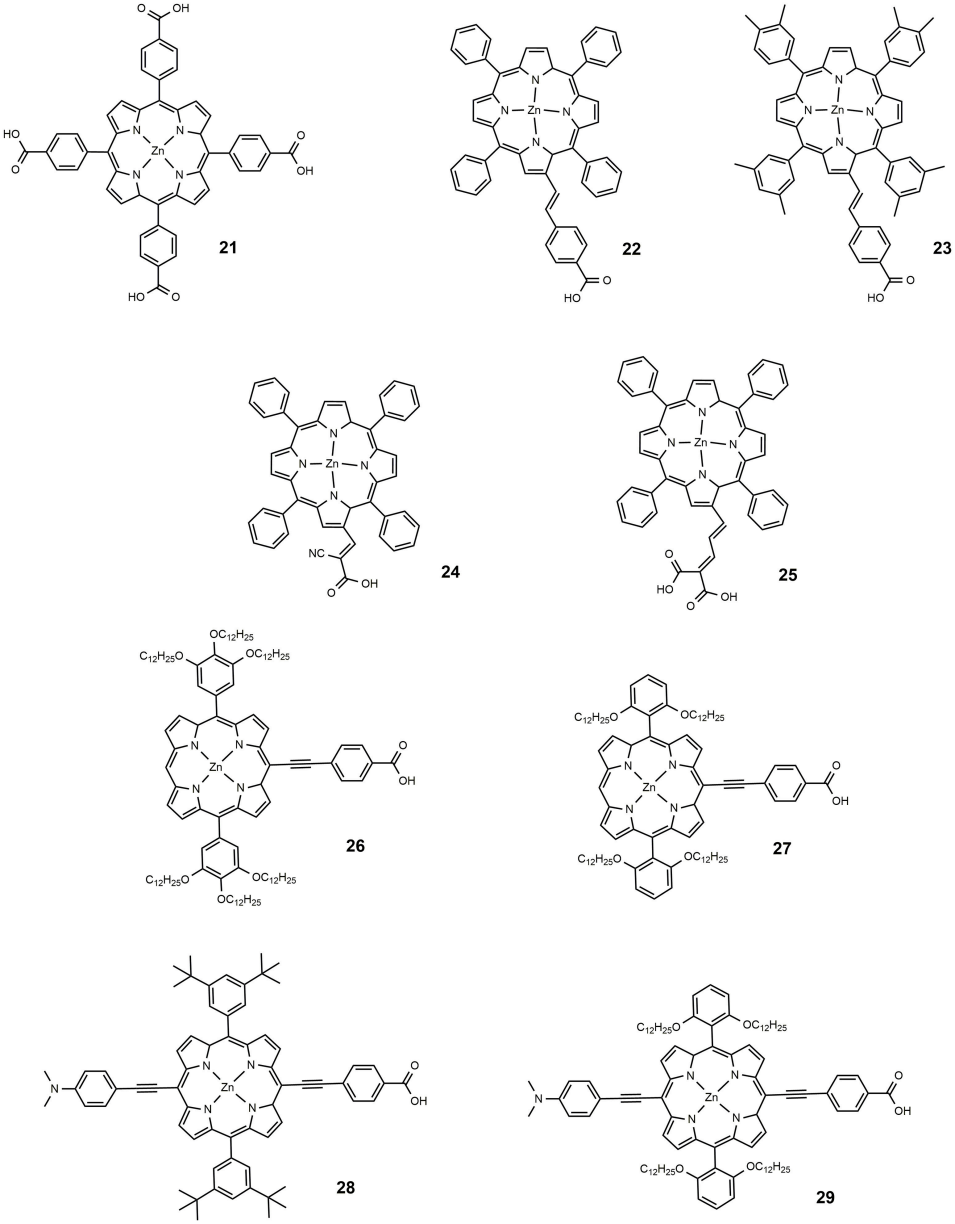
Chemical structure of dyes 21–29.

The second important class of metal containing DSSC sensitizers is represented by porphyrin-based dyes. The optical behavior of porphyrin is characterized by two intense optical absorptions, the Soret band between 400 and 500 nm and the Q-band, between 550 and 750 nm. The functionalization of the macrocycle constituting the structure of porphyrin allows to tune these two bands in sucha way that panchromatic dyes suitable for DSSC applications can be obtained. In 2000 a very important study describing the charge transfer process between tetrakis(4-carboxyphenyl)porphyrin (dye **21**, Figure 4) and TiO_2_ (Tachibana et al., 2000) revealed a very fast electron injection kinetic and similar to that of ruthenium based photosensitizers. This paper paved the way for the design and study of various porphyrin-based molecules as sensitizers for DSSC.

An important drawback of porphyrin dyes is the tendency to aggregate on the TiO_2_ surface, leading to a self-quenching of the adsorbed photon before the desired electron injection in the semiconductor. To reduce aggregation co-adsorbates (Zhang and Cole, 2017) are typically employed with the aim of keeping the molecules physically separate. Among the most popular co-adsorbates it is worth mentioning the chenodeoxycholic acid (CDCA) (Lee et al., 2009) the hexadecylmalonic acid (HDMA) (Wang et al., 2003a) and the dineohexyl bis(3,3-dimethylbutyl)phosphinic acid (DINHOP) (Wang et al., 2009). The use of a long alkyl chain to functionalize the macrocycle core helps to avoid aggregation and protects TiO_2_ from undesired interactions with electrolyte.

In 2004, Nazeeruddin proposed a series of tetraphenyl Zn porphyrins, substituted in the β-pyrrolic position with a styril carboxylic acid (Nazeeruddin et al., 2004). The breaking of porphyrin symmetry, with the phenyl moiety acting as donor and the carboxylic acid as acceptor, determined a red shift of Q-band; the dyes **22** and **23** (shown in Figure 4), used as DSSC sensitizers, provided an efficiency of 4.1 and 4.8%, respectively. The increased efficiency of the latter was associated with the bulkier xylyl group employed which to some extent hindered the aggregation of the sensitizer on the surface of TiO_2_, one of the main drawbacks of porphyrinic dyes. The use of different anchor groups was also explored: in 2005, a new porphyrin sensitizer was reported, **24** (Wang et al., 2005b) in which the macrocycle was functionalized with a cyanoacrylic group (see Figure 4). The insertion of the electron withdrawing cyano group on the carboxylic moiety determined a red shift of the optical absorption and, at the same, an increased electron density on the anchor group, desirable for the electron injection process. An efficiency of 5.2% was found for a dye **24** sensitized device.

In 2007, a series of novel porphyrinic sensitizers was reported containing malonic acid as binding group: all dyes allowed an efficiency of more than than 5% (Campbell et al., 2007); dye **25** (Figure 4) was the most active sensitizer in that paper, with an efficiency of 7.1%.

The functionalization of the porphyrin macrocycle with the anchoring group can also take place in *meso* position. In 2011, Lin developed alkoxyphenyl-substituted porphyrins (dyes **26–29**, Figure 4) in which an ethynyl-benzoic acid was placed in *meso* (Chang et al., 2011). All sensitizers were efficiently used in DSSC devices giving efficiency from 4.78 to 10.17%. The authors suggested that the functionalization with long alkoxy-chains in the *ortho* position of the phenyl group had the effect of wrapping the porphyrin core, limiting the aggregation and delaying the charge recombination between the injected electrons and the redox couple (iodide/triiodide).

The most performing dyes reported in that paper, dye **28** and **29**, were characterized by the presence of a dimethylanilino-donor group: the concept of *push-pull* porphyrins, where the macrocycle acts as π-spacer between a strong electron donor group and an electron acceptor group, was found very fruitful in order to extend optical absorption and harvest a larger fraction of solar photons. Dyes **28** and **29** have absorption maxima at 672 and 667 nm (with high ε values, exceeding 65,000) and DSSC based on them, consequently, are characterized by exceptional J_SC_ values (see Table 2).

**Table 2:** Photovoltaics parameters of DSSC sensitized with porphyrin-based sensitizers 22–41.

**Dye**	**Code**	**J_SC_**	**V_OC_**	**FF**	**η**	**λ_MAX_(nm)/ε × 10^−3^**	**References**
		**(mA·cm^−2^)**	**(V)**		**(%)**	**(M^−1^·cm^−1^)**	
22	ZnTPSCA	8.86	0.654	0.71	4.1	436/225, 565/20.4	Nazeeruddin et al., 2004
23	ZnTXPSCA	9.70	0.660	0.75	4.8	–	Nazeeruddin et al., 2004
24	GD1	13.5	0.566	–	5.2	455/153, 571/12.7, 620/11.9	Wang et al., 2005b
25	Zn1	14.0	0.680	0.74	7.1	442/155, 570/16.6, 610/9.29	Campbell et al., 2007
26	LD11	9.735	0.674	0.728	4.8	441/437, 569/18.2, 616/24.0	Chang et al., 2011
27	LD12	13.235	0.741	0.758	7.43	442/437, 569/19.1, 619/26.3	Chang et al., 2011
28	LD13	18.438	0.697	0.727	9.34	458/275, 672/95.5	Chang et al., 2011
29	LD14	19.167	0.736	0.721	10.17	459/251, 667/66.1	Chang et al., 2011
30	YD1	13.05	0.712	0.703	6.54	430/616, 565/20.7, 605/14.7^a^	Lu et al., 2009
31	YD2	13.68	0.711	0.695	6.76	–	Lu et al., 2009
31	YD2	18.6	0.77	0.764	10.9	444/217, 589/10.8, 648/33.7^b^	Bessho et al., 2010
32	ZnPBAT	19.33	0.719	0.724	10.1	433/75, 460/61.5, 596/7.03, 661/17.1	Kurotobi et al., 2013
33	LAC-3	12.67	0.67	0.64	5.44	464/100, 501/148, 655/64.6	Lin et al., 2010
34	LD4	19.627	0.711	0.721	10.06	464/417, 672/95.5	Wang et al., 2011
35	YD2-o-C8	17.3	0.965	0.71	11.9	448/212, 581/12, 645/31	Yella et al., 2011
35	YD2-o-C8^c^	17.66	0.935	0.74	12.5	–	Yella et al., 2011
37	GY-50	18.53	0.885	0.773	12.75	453/119, 538/11, 665/53	Yella et al., 2014
38	SM315	18.10	0.91	0.78	13.0	440/105, 454/117, 581/12, 668/53	Mathew et al., 2014
39	ZnP^d^	19.36	0.735	0.71	10.1	456(167), 666 (47)	Chang et al., 2013
41	XW11^e^	20.33	0.76	0.74	11.5	465/161.8, 622/14.5, 683/85.7	Xie et al., 2015

a *Optical properties from Lee et al. (2009)*.

b *Optical properties from Hsieh et al. (2010)*.

c *Cosensitized with dye 43*.

d *Cosensitized with dye 40*.

e *Cosensitized with dye 42*.

**Figure 5:**
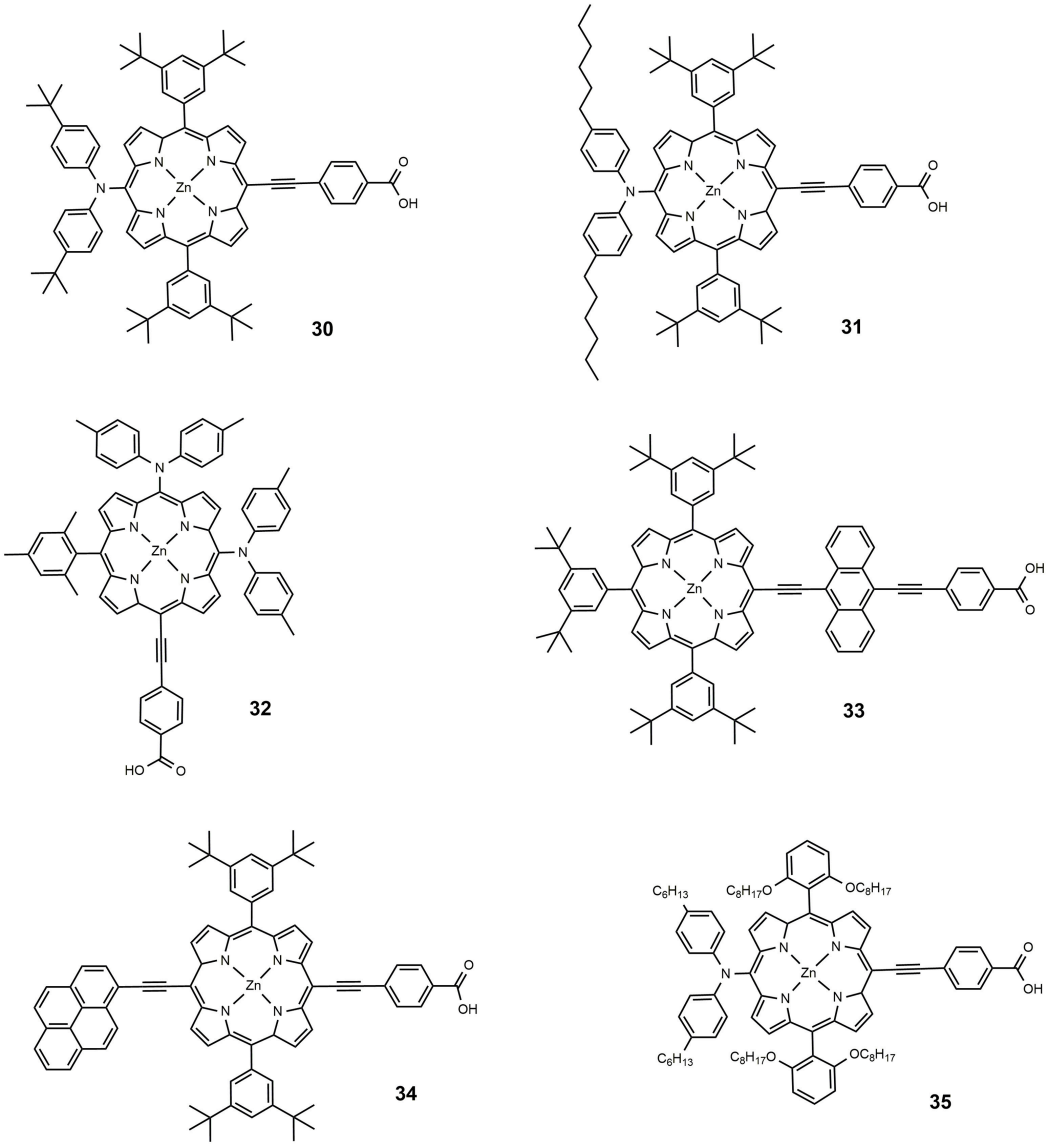
Chemical structures of dyes 30–35.

In 2009, a series of Zn porphyrins with a diarylamino group as strong electron donor substituent and ethynyl-carboxy moiety as acceptor, were prepared and studied as DSSC photosensitizers (Lu et al., 2009): compared to similar diarylamino-free systems, a broadening of the Soret band and a remarkable red shift of the Q band characterized the optical absorption of the new molecules, **30** and **31**, whose molecular structure is reported in Figure 5. An efficiency of 6.8% was achieved for the devices sensitized with **31**, slightly higher than the efficiency reported for **30** (6.5%). In a following paper, an efficiency approaching 11% was reported using **31** (Bessho et al., 2010), as a consequence of device optimization. This paper represented the first example of porphyrin based DSSC that exceeded 10% efficiency. The introduction of various diarylamino groups into the porphyrin structure was also investigated by Imahori group (Imahori et al., 2010; Kurotobi et al., 2013). With the increase in the number of the diarylamino groups, the Soret band widens and slightly blue-shifts, while a new band appears at 450–500 nm and the Q band shifts toward longer wavelengths. The optimization of the molecular structure led to sensitizer **32**, which afforded devices with an efficiency of 10.1% (Kurotobi et al., 2013).

The effect of introducing additional π-chromophore into the molecular backbone was also investigated. By systematically introducing different acenes into the porphyrin core, Lin et al. (2010) have shown that the absorption has broadened and red-shifted with the increase in the acene size (from benzene to pentacene). The best result was obtained for anthracene-containing porphyrin (dye **33**, Figure 5) with an efficiency of 5.4%, the authors suggested the role of charge delocalization between the porphyrin core and the anthracene group. Better results were obtained by the same group by introducing the π-chromophore at the *meso*-position, opposite to the anchoring group (Wang et al., 2011), with an outstanding efficiency of 10.1% obtained with the sensitizer **34**: the dye showed red shifted absorption (Q band centered at 676 nm) and suitable electrochemical properties.

A milestone paper in the field of porphyrin DSSC photosensitizers was reported in 2011 (Yella et al., 2011): dye **35** combines many of the previously studied concepts such as the use of diaryl-amino moiety to obtain a *push-pull* system and the functionalization in *o*-position with long alkoxy tails that prevent the aggregation of molecules and protect the TiO_2_ from direct interaction with the electrolyte. The real breakthrough was the use of a different redox mediator from the classical iodide/triiodide couple: using Co^II/III^ tris(bipyridyl) complex in the electrolyte solution, an efficiency of 11.9% was achieved compared to 7.6% obtained with I^−^/I${}_{3}^{-}$ redox couple. The lower value of the redox potential for Co(II)/Co(III) couple produced a very high V_OC_, 0.965 V (see Table 2). At the same time a considerable current density was observed with J_SC_ equal to 17.5 mA/cm^2^.

**Figure 6:**
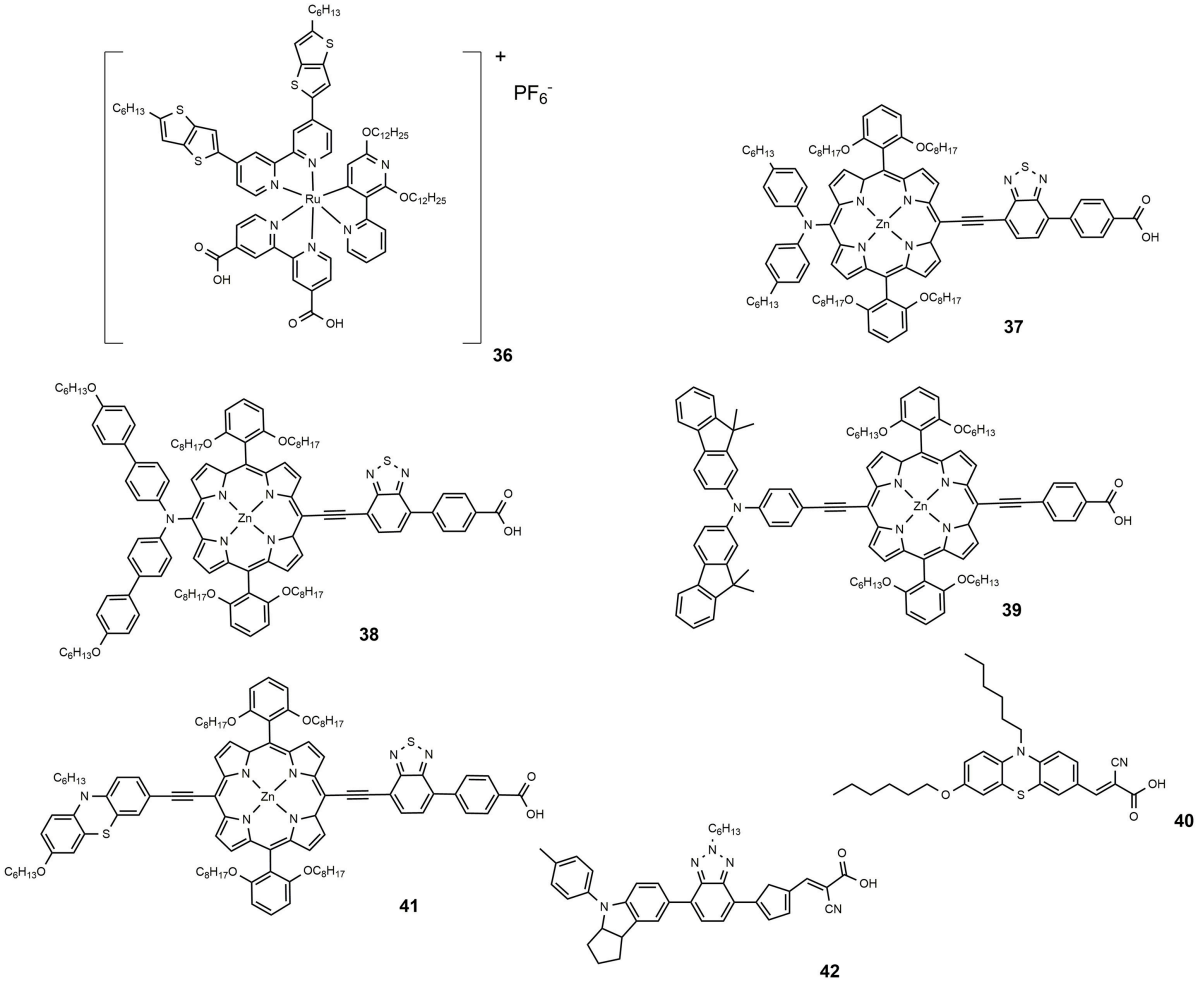
Chemical structures of dyes 36–42.

Co-polypyridyl complexes are bulkier than the iodide/triiodide couple and in this sense, the effect of the alkoxy tails to hinder the redox mediator approach to the TiO_2_ is more pronounced. Cobalt-based electrolytes have been widely investigated in the field of DSSC since that paper. It is interesting to notice that the classical Ru-based sensitizers containing thiocyanate ligands do not perform well with cobalt electrolytes due to the direct interaction between ruthenium dyes and the cobalt redox species which reduced the charge injection from the triplet state of the dyes to the titanium oxide and increased the electron recombination process with the cobalt redox species (Omata et al., 2015). The design of SCN-free cyclometalated ruthenium dyes is the most promising strategy to exploit the favorable redox properties of cobalt complexes mediators: in 2016 a novel thiocyanate-free Ru-based sensitizer (dye **36**, Figure 6) was prepared and used in combination with a cobalt-based electrolyte (Aghazada et al., 2016): the DSSC sensitized with this cyclometalated dye provided a very promising efficiency of 9.4% with a VOC of 0.845 V.

In 2014, a new porphyrin sensitizer appeared (**37**, see Figure 6), a *push-pull* system characterized by the insertion of a strong electron withdrawing group (Yella et al., 2014). An impressive efficiency of 12.75% was obtained using a cobalt-based electrolyte. A slight structural modification of **37**, consisting in the functionalization with alkoxy tails of the phenyl groups of the diarylamino donor group, led to **38**, which afforded an efficiency of 13% (Mathew et al., 2014).

One of the main drawbacks of porphyrin-based sensitizers lies in a reduced absorption between 500 and 600 nm. The use of two or more different sensitizers with complementary absorption properties is an effective approach to improve the power conversion efficiency of the DSSC device. Co-sensitization have a double beneficial effect on DSSC operation: on the one hand, by increasing the amount of harvested solar photons an increase of photo-generated current is observed. On the other hand, a better coverage of the semiconductor surface reduced the detrimental charge recombination phenomena between the injected electron and the redox couple (Krishna et al., 2017). Co-sensitization can be achieved through two different processes: In the cocktail approach, co-sensitization is performed by mixing two sensitizers solutions with a specific molar ratio, while the stepwise approach is performed by sequential adsorption of the two different dye solutions. The second process was employed by Chen et al. in making co-sensitized DSSC devices with porphyrin-based dye **39** and phenothiazine-based metal-free **40** (Chang et al., 2013). An efficiency value of 10.1% was achieved, higher that that obtained with only **40** (8.2%) or only **39** (7.4%). The deposition of the smaller molecule **40** after the larger one, **39**, determined, according to the authors, an optimal surface coverage of TiO_2_ surface and explained the superior performance of the device. Co-sensitization, achieved by sequential adsorption, of phenothiazine-containing porphyrin sensitizer **41** and metal-free dye **42** was performed in 2015 (Xie et al., 2015). The two sensitizers presented complementary optical absorptions, with **42** showing an intense and broad band centered at around 510 nm and **41** showing the typical Soret band (465 nm) and Q band (683 nm). The co-sensitized device afforded an efficiency of 11.5%, considerably higher than the efficiency shown by the device sensitized only with **40** (7.8%).

**Figure 7:**
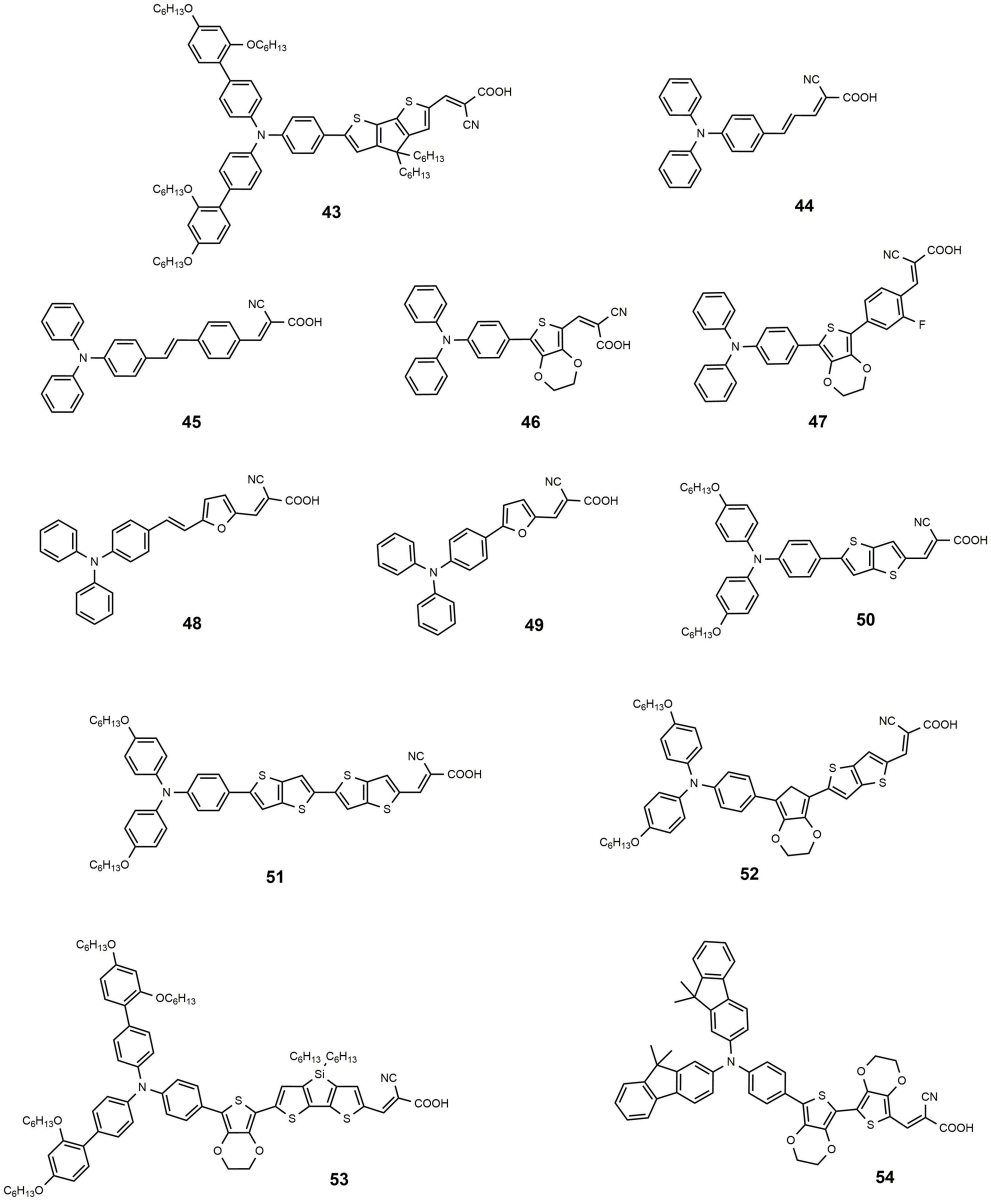
Chemical structures of dyes 43–54.

This efficiency value represents the record for non-ruthenium DSSCs using the I^−^/I${}_{3}^{-}$ redox couple. A further improvement of the previously discussed dye **35** was obtained by co-sensitization with dye **43** (see Figure 7). The latter is characterized by a complementary adsorption with a maximum at 532 nm in THF (53,000 M^−1^ cm^−1^) that coincides with the minimum in the IPCE spectral response of the **35**. Through co-sensitization an efficiency value of 12.3% was achieved (Yella et al., 2011).

### Metal-free sensitizers

Metal-free organic dyes represent the third main class of photosensitizers for DSSC. With respect to ruthenium-based sensitizers they feature a more straightforward tunability of the chemical structure by exploiting the well-established synthetic strategies applied in the chemistry of dyes: the modulation of the chemical properties results, in turn, in a great variability of the optical properties so that sensitizers with broad spectral absorption can be obtained. Metal-free sensitizers are also typically characterized by large molar extinction coefficients and this aspect is particularly relevant for electrolyte-free solid-state DSSC, where a semiconductor thickness not exceeding 2–3 mm is desirable to allow an optimal charge collection (Mishra et al., 2009).

The prototypal metal-free sensitizer is based on a push-pull structure D-π-A where an electron donor (D) group is linked to an electron acceptor (A) through a π-conjugated bridge (π). Typically, the electron acceptor also acts as the anchoring group that binds the dye to the semiconductor surface. This type of structure is favorable because it facilitates the separation of photoinduced charge: upon photoexcitation, a shift of electron density occurs from the donor part of the molecule (where the HOMO is mainly localized) toward the electron acceptor side (where the LUMO is mainly localized), thus facilitating the electron injection into the conduction band of TiO_2_ and, at the same time, hindering the deleterious process of charge recombination between semiconductor and oxidized molecule. The rational design of novel metal-free sensitizers has been focused on investigating different electron donors and π-linkers and, to a lesser extent, electron-withdrawing anchoring moieties. The aim of these research efforts was to obtain systems with adequate optical absorption, which extend in particular in the NIR area of the spectrum, well-aligned electronic levels to minimize losses and high stability during device operation (Ahmad et al., 2013). It is worth noticing that, as in the case of porphyrinic dyes, the tendency to aggregation is an important issue and to achieve high efficiency the use of co-adsorbates is often mandatory (Zhang and Cole, 2017).

Among the donor units, triphenylamine (TPA) and derivatives are by far the most studied (Ahmad et al., 2013). The triphenylamine donor was introduced for the first time in 2004 (Kitamura et al., 2004): dye **44** (see Figure 7) was employed as photosensitizer in a classical DSSC device based on iodine electrolyte and afforded a promising efficiency of 5.30%. The introduction in the conjugated bridge of a stilbenyl unit led to dye **45**, which sensitized a TiO_2_ DSSC with efficiency of 5.73% (Teng et al., 2010). The electron rich thiophene rings have been widely investigated as a component of the conjugated bridge of the metal-free DSSC sensitizers. In 2008 Liu proposed a series of TPA dyes containing thiophene derivatives in molecular backbone (Liu et al., 2008). Among these dyes the best efficiency was demonstrated by sensitizer **46** containing 3,4-ethylendioxythiophene group (EDOT), as a consequence of a broad spectral response and high molar extinction coefficient (24,500 M^−1^ cm^−1^): this dye sensitized a device with a very interesting efficiency of 7.30%. In a subsequent work, the same group prepared new TPA dyes containing EDOT groups coupled to fluorine-functionalized phenyl groups: dye **47** showed an excellent performance as DSSC photosensitizer, achieving an efficiency of 8.22% (Chen et al., 2011). In the same paper, this dye was efficiently used as a photosensitizer in a solid-state DSSC with an efficiency of 4.53%.

In 2009 Lin et al. studied the effect of replacing the thiophene ring with the furan ring: considering the smaller resonance energy of the furan with respect to the thiophene ring, the former should in theory provide a more effective conjugation in the π-bridge of the dyes. A good performance was reported for dye **48** and **49** with efficiency respectively of 7.36 and 6.30% (Lin et al., 2009). In the same paper a comparison was made between **49** and its thiophene congener: the latter showed a lower efficiency of 6.09% suggesting that the furan ring could be in effect a good alternative to thiophene in conjugated bridge of the dye.

As already discussed for Ru-based and porphyrin-based photosensitizers, the use of bulky alkyl or alkoxy tails is a useful strategy to improve the efficiency of the DSSC by creating a sort of passivation around TiO_2_ surface that prevents undesired charge recombination reaction at the semiconductor/electrolyte interface. In this regard, TPA functionalized with dihexyloxy (DHO) represents one of the most efficient donors in metal-free sensitizers for high performance DSSCs. Hexyloxy tails also have the effect to increase the electron donor ability of TPA group. In 2009 Zhang reported some new phosensitizers characterized by the presence of DHO-TPA group (Zhang et al., 2009a,b). Dyes **50** and **51** contain a thienothiophene and a bis-thienothiophene group, respectively, in the molecular backbone (Zhang et al., 2009a). The insertion of these electron-rich heterocycles improves the light harvesting capacity of the dyes, leading to molar extinction coefficients of 42,000 (at 516 nm) for **50** and 47,000 (at 524 nm) for **51**. The devices sensitized with **51** provided an efficiency slightly higher than those sensitized with **50** (8.02 vs. 7.54%).

The replacement of one thienothiophene group of **51** with EDOT group produces dye **52** (Zhang et al., 2009b): this structural modification gives rise to a significant red-shift of optical absorption with the maximum absorption wavelength shifting to 552 nm. Device sensitized with **51** provided an impressive efficiency of 9.8%. The same dye was used as sensitizer in a solvent-free DSSC which afforded an efficiency of 8.1% and an excellent stability: after 1,000 h under one sun soaking the device still retained 96% of its initial performance.

The dye **43**, reported in 2011 (Tsao et al., 2011b) is characterized by DHO-TPA donor group; the insertion in its molecular skeleton of a cyclopentadithiophene electron-rich moiety determined a red shifted absorption (absorption maximum at 538 nm with ε of 53,000 M^−1^ cm^−1^). By using a cobalt base electrolyte, a DSSC device sensitized with **43** provided an efficiency of 10.3% (Tsao et al., 2011a). In a different paper the same dye was used to sensitize a solid-state DSSC, employing a copper based hole transporting material and a record efficiency (for solid state DSSC) of 11% was obtained (Cao et al., 2017). The introduction of an EDOT unit and the replacement of cyclopentadithiophene ring with dithienosilole unit afforded the dye **53**, shown in Figure 7: the dye is characterized by a very high molar extinction coefficient (57,500 at 493 nm) and was used as DSSC sensitizer with an efficiency of 10.3% by using iodine based electrolyte (Zeng et al., 2010).

**Figure 8:**
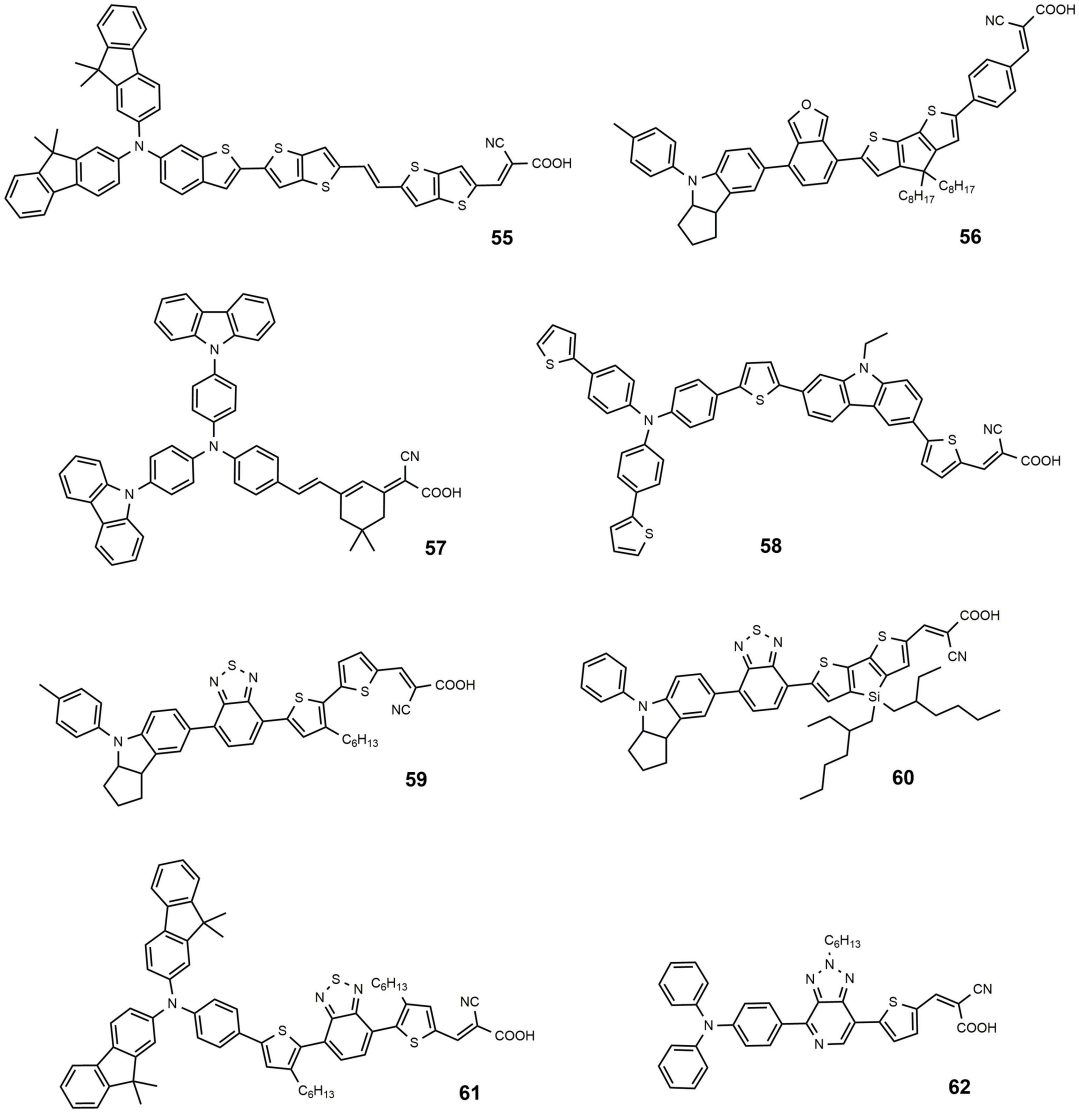
Chemical structures of dyes 55–62.

A fluorene-based variation of TPA donor moiety was used by the Gratzel group in 2009 (Xu et al., 2009): the dye **54** (Figure 7) showed an efficiency of 8.32%. The same donor was used by Choi et al. in the dye **55** reported in Figure 8: this dye featured a very high molar extinction coefficient (85,000 M^−1^cm^−1^ at 490 nm, as shown in Table 3) and the device sensitized with it showed a J_sc_ of 17.61 mA/cm^2^ and an overall efficiency of 9.1%. In the same paper the dye **55** was used as sensitizer in a device based on a solvent-free ionic liquid which provided a relevant efficiency of 7.9% and a very high operational stability under light soaking at 60°C for 1,000 h. Derivatives of indoline were also explored as electron donors with excellent results: dye **56**, shown in Figure 8, containing an indoline donor group and a cyclopentadithiophene spacer, provided devices with efficiency up to 9.03%.

**Table 3:** Photovoltaics parameters of DSSC sensitized with metal free sensitizers 43–62.

**Dye**	**Code**	**J_SC_**	**V_OC_**	**FF**	**η**	**λ_MAX_(nm)/(ε × 10^−3^/**	**References**
		**(mA·cm^−2^)**	**(V)**		**(%)**	**(M^−1^·cm^−1^)**	
43	Y123	15.9	0.910	0.71	10.3	532/53.0	Tsao et al., 2011a
44	–	11.1	0.73	0.66	5.30	417/25.0	Kitamura et al., 2004
45	TPC1	10.39	0.70	0.78	5.73	438/37.6	Teng et al., 2010
46	LJ1	15.50	0.69	0.68	7.30	426/24.5	Liu et al., 2008
47	LJB-F0	15.58	0.79	0.67	8.22	420/42.2	Chen et al., 2011
48	–	16.59	0.69	0.64	7.36	469/33.0	Lin et al., 2009
49	–	14.16	0.68	0.66	6.30	456/33.5	Lin et al., 2009
50	C206	13.9	0.73	0.74	7.54	516/42.0	Zhang et al., 2009a
51	C211	15.2	0.72	0.73	8.02	524/47.0	Zhang et al., 2009a
52	C217	16.1	0.80	0.76	9.80	552/–	Zhang et al., 2009b
53	C219	17.9	0.730	0.73	10.3	493/57.5	Zeng et al., 2010
54	C205	15.68	0.746	0.711	8.32	544/38.5	Xu et al., 2009
55	JK113	17.6	0.710	0.72	9.10	490/85.0	Choi et al., 2010
56	WS69	19.39	0.696	0.67	9.03	568/48.5	Zhang et al., 2017
57	S4	13.8	0.630	0.69	6.02	480/25.0	Ning et al., 2008
58	TTC104	13.3	0.774	0.58	6.37	425/27.1	Zhang et al., 2013
59	WS-9	18.00	0.696	0.72	9.04	536/20.8	Wu et al., 2012
60	B87	20.28	0.724	0.68	10.26	562/47.4	Gao et al., 2016
61	JK69	14.98	0.770	0.74	8.19	468/19.3	Kim et al., 2008
62	NPT5	14.90	0.720	0.74	7.92	486/54.8	Chaurasia et al., 2015b

Functionalization of TPA donor with an electron-rich carbazole unit offered an interesting approach based on D-D-π-A structure: this kind of structure has been shown to provide a better photochemical and thermal stability than the simple *push-pull* structure. The dye **57** belongs to this class and afforded a device with efficiency of 6.02% (Ning et al., 2008). The dye **58** is a further example employing a thiophene-substituted tryarilamine as donor: the device sensitized with this dye produced an efficiency of 6.37% (Zhang et al., 2013).

A different approach that has been explored is based on the insertion of an electron-poor moiety into the π-bridge, thus creating a D-A-π-A structure. This type of structure has recently become particularly attractive, especially to achieve high extinction coefficients, broaden the absorption, and improve the photo/thermal stability of organic sensitizing dyes (Wu and Zhu, 2013). The incorporation of a benzothiadiazole group in the π-bridge was explored by several groups with interesting results. The structure of dye **59** (see Figure 8) is characterized by the insertion of an electron-poor benzothiadiazole group between an indoline donor and a bithiophene bearing the final acceptor (anchoring) cyanoacrylic moiety (Wu et al., 2012). A red-shift of the absorption (maximum at 536 nm) and an efficiency of 9.04% of the DSSC were obtained, with a particularly high J_SC_ (18.9 mA/cm^2^). By keeping an indoline donor and an auxiliary benzothiadiazole acceptor group, dye **60** was characterized by the use in the π-bridge of 4-bis(2-ethylhexyl)-4H-silolo[3,2-b:4,5-b0]dithiophene (see Figure 8), which was found to work very effectively in reducing dye aggregation (Gao et al., 2016). A device with a very high efficiency of 10.26% was obtained using this dye. The cell showed an outstanding stability, retaining 95% of the initial efficiency after continuous light soaking for 1,000 h at 60°C.

**Figure 9:**
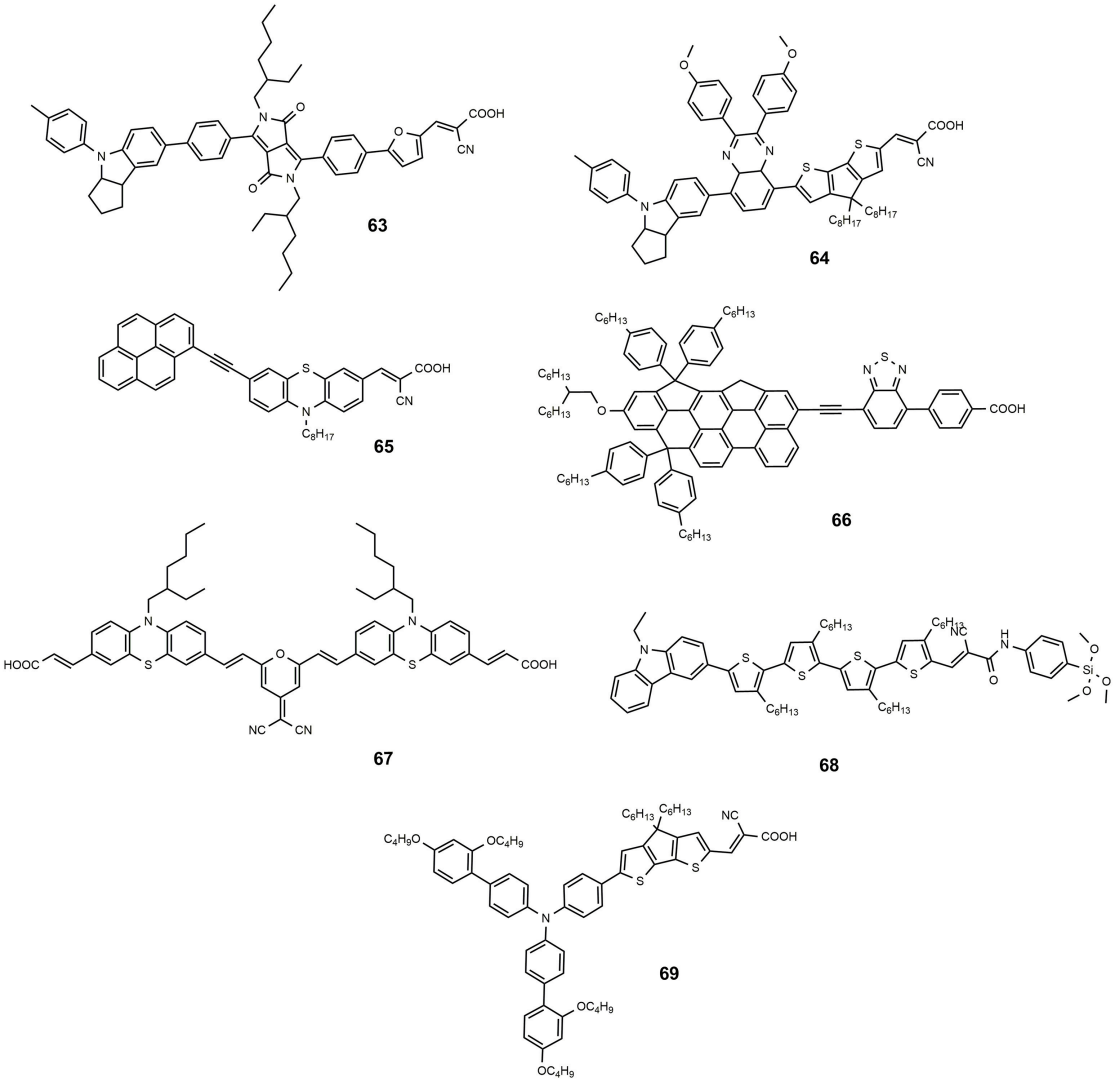
Chemical structures of dyes 63–69.

Dye **61** also contains a benzothiadiazole group in the molecular framework with a fluorene derivative of TPA as donor: an efficiency of 8.19% was found together with a relevant stability of the device which retained 90% of its performance after 1,000 h light soaking (Kim et al., 2008). Various derivatives of benzothiadiazole were also explored by Lin group (Chaurasia and Lin, 2016). Dye **62**, containing a pyridal[2,1,3]benzotriazole, was investigated as DSSC photosensitizer affording an efficiency of 7.92% (Chaurasia et al., 2015b); the use of a weaker electron acceptor compared to benzothiadiazole was thought to exert a weaker charge trap effect, thus favoring the migration of the electron toward the anchoring cyanoacrylic group. The dye **63**, whose structure is reported in Figure 9, belongs to the class of D-A-π-A sensitizers as well: it is characterized by the presence of a diketopyrrolopyrrole electron withdrawing group in the molecular backbone and an indoline type donor group (Qu et al., 2012). Its absorption is at 526 nm with a molar extinction coefficient of 4.6·10^4^ and it gave place to devices with an efficiency of 7.43% (see Table 4) and excellent stability (insignificant variations of electrical parameters of the device after 1,000 h at 60°C under light soaking). The dye **64** is characterized by the quinoxaline acceptor group and showed a maximum absorption maximum at 555 nm (ε = 63200). By using a volatile electrolyte, a device with an efficiency of 9.83% was obtained. In the same paper a quasi-solid DSSC, based on a polymer gel electrolyte, was prepared and showed an impressive efficiency of 8.76%.

**Table 4:** Photovoltaics parameters of DSSC sensitized with metal free sensitizers 62–71.

**Dye**	**Code**	**J_SC_**	**V_OC_**	**FF**	**η**	**λ_MAX_(nm)/ε × 10^−3^**	**References**
		**(mA·cm^−2^)**	**(V)**		**(%)**	**(M^−1^·cm^−1^)**	
63	YCD01	13.4	0.760	0.73	7.43	526/46.0	Qu et al., 2012
64	IQ22	18.36	0.748	0.72	9.83	555/63.2	Wang et al., 2016
65	–	24.2	0.846	0.590	12.1	438/23.5	Nagarajan et al., 2017
66	C293	17.28	0.974	0.747	12.6	552/–	Wang et al., 2017
67	CB1	6.7	0.659	0.64	2.8	478/62.0	Maglione et al., 2016a
68	ADEKA	15.6	1.036	0.774	12.5	498/43.2	Kakiage et al., 2014
68	ADEK^a^	18.27	1.014	0.771	14.3	498/43.2	Kakiage et al., 2015
70	DPP17	17.9	0.761	0.74	10.1	602/69	Yum et al., 2013
71	R4	17.25	0.852	0.754	11.1	613/85.5	Ren et al., 2018
72	R6	19.69	0.850	0.754	12.6	631/81.8	Ren et al., 2018

a *Cosensitized with dye 69*.

The use of a rigid aromatic system in the molecular backbone has been extensively studied in order to increase the overall planarity and favor a strong electrical communication between the donor and the acceptor parts of the molecule (Chaurasia et al., 2015a). The dye **65** has a quite simple structure based on a donor phenothiazine group linked to a classic cyanoacrylic anchor group. The presence of pyrene at the 7-position of the phenothiazine ring and the alkynyl bridge not only ensures a hydrophobic periphery, hindering the desorption induced by water, but also results in an extended conjugation, improving the absorption capacity and thereby resulting in good photovoltaic performance. A giant J_SC_ value was afforded by DSSC device sensitized with this dye (24.2 mA/cm^2^, see Table 4) which resulted in an overall efficiency exceeding 12% (Nagarajan et al., 2017). An even higher performance was shown by the dye **66**. The dye contained a rigidified N-annulated benzoindenopentaphene (a nonacyclic aromatic hydrocarbon), functionalized with multiple solubilizing groups. This fragment was coupled through an ethynyl group to a benzothiadiazole-benzoic acid moiety. An outstanding efficiency of 12.6% was obtained for the DSSC based on this dye (see Table 4) and this value represents, to date, the higher efficiency for a DSSC sensitized with a single metal-free dye (Wang et al., 2017).

Organic sensitizers, characterized by the presence of two anchoring groups to increase the of the dye toward leaching (Meier et al., 2017), have also been studied. This kind of systems can be obtained by bridging two mono-anchoring dye molecules (Sirohi et al., 2012) with the effect of increasing molar absorption coefficients and reduce aggregation. Following this approach, our group proposed a series of double anchored dyes, based on a pyran core functionalized with several electron acceptor moieties and symmetrically coupled to carbazole or phenothiazine donor moieties (Maglione et al., 2016a,b). The functionalization of a same molecular fragment with different electron acceptor groups allows a wide tuning of the optical properties so that the dyes cover most of the visible spectrum. A very high molar extinction coefficient was found, of up to 1.0·10^5^ M^−1^·cm^−1^. The overall efficiency of the devices sensitized with these dyes reached a maximum value of 2.8% for the dye **67**. The devices showed however a very high operational stability: after 1,000 h of thermal curing at 85°C no variation of the main electrical parameters was observed. The non-exceptional efficiency was ascribed to a non-ideal electronic structure with the electron density moving toward the core of the dye on photoexcitation: this behavior suggests a more convenient use of these dyes as photosensitizers in p-type DSSC where they, indeed, were tested showing promising results (Bonomo et al., 2017, 2018).

As described in this paragraph, the anchoring group of the typical metal-free photosensitizer is a cyanoacrylic moiety which coincides with the electron acceptor part of the dye. Although many other anchoring groups have been tested, as pyridine, phosphonic acid, benzoic acid (Zhang and Cole, 2015) or tetrazole (Massin et al., 2014) and triazole derivatives (Centore et al., 2015), the best performing organic sensitizers, until recently, contained a cyanoacrylic-like anchoring group. In the last year, however, the Kakiage group investigated the alkoxysilyl moiety as a possible alternative anchoring group and prepared new photosensitizers with outstanding performances. The dye **68** was employed in a DSSC affording an efficiency up to 12.5% (Kakiage et al., 2014). The result was mostly attributed to the strong adsorption properties of **68** to the TiO_2_ electrode and shows the validity of silyl-anchor dyes as photosensitizers for DSSCs. The same dye was used in a co-sensitized DSSC with another dye, **69**, producing an efficiency of up to 11.1% when a iodine-based electrolyte was used and of up to 14.3% with a cobalt-based electrolyte (Kakiage et al., 2015). This value represents the current efficiency record for organic-based DSSC.

**Figure 10:**
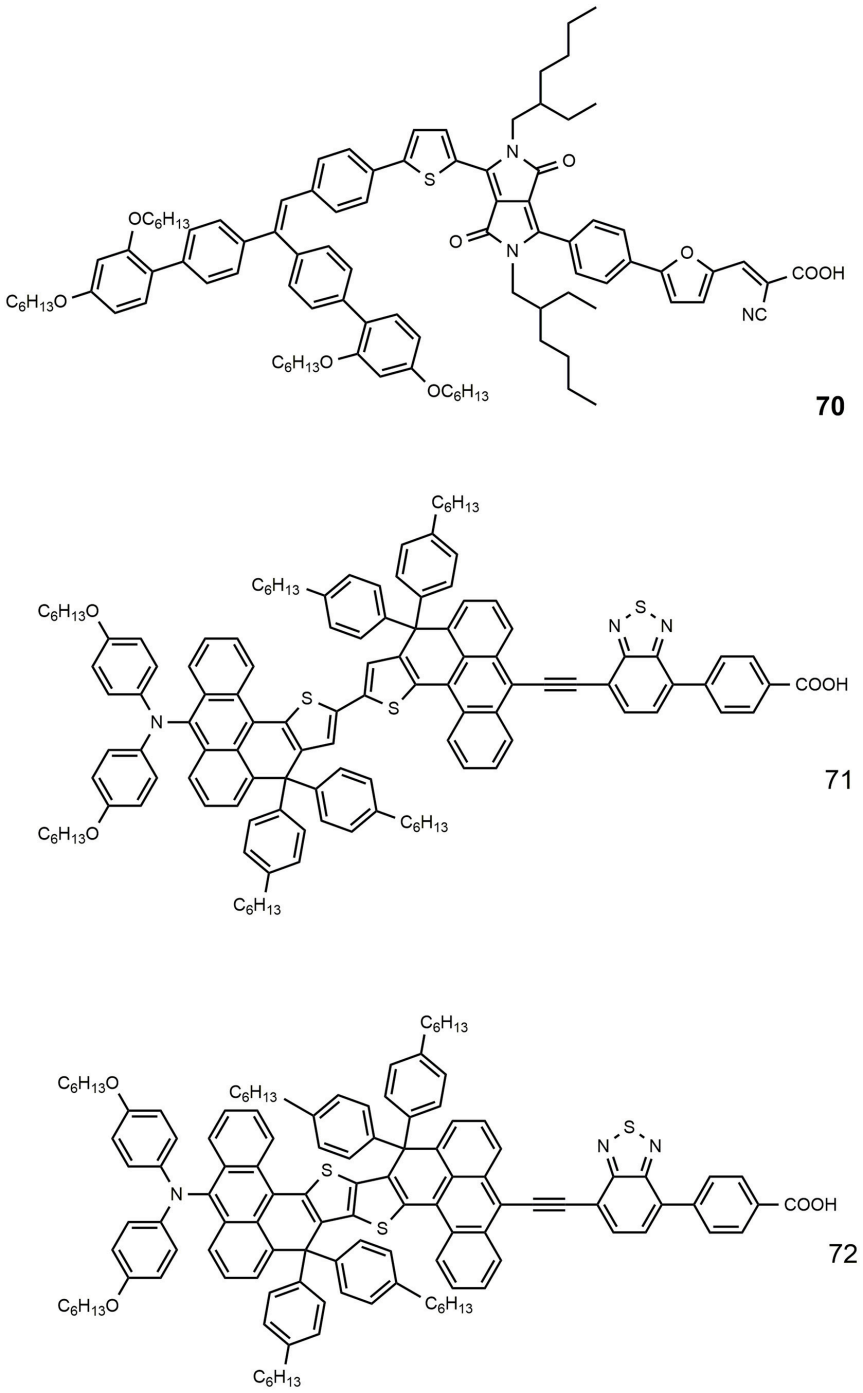
Chemical structures of dyes 70–72.

The number of highly efficient DSSCs that use blue photosensitizers is rather limited due to a great tendency to aggregation and mismatched energy levels for electron injection (typically, the absorption at higher wavelengths is the result of the LUMO stabilization). Figure 10 shows the chemical structure of some efficient blue dyes. The dye **70** is based on an asymmetrical diketopyrrolopyrrole derivative: it is characterized by an intense absorption with peak at 603 nm and sensitized a DSSC with efficiency of 10.1% (Yum et al., 2013). An even higher efficiency was reported for the dyes **71** and **72**, characterized by an anthracene-based rigidified aromatic unit (double) in the π-bridge: the two dyes afforded outstanding efficiency of, respectively, 11.1 and 12.5% (Ren et al., 2018) in combination with a cobalt-based electrolyte.

## Summary and perspectives

The purpose of this Review was to provide an overview of the development of the main classes of DSSC photosensitizers and of the design strategies that have been established for the optimization of their performance in real devices.

Ru(II) polypyridyl complexes represent the first generation of DSSC sensitizers to be investigated and the huge amount of studies on these systems has produced useful guidelines for the design of other types of dyes. The molecular engineering strategies employed to optimize Ru-based photosensitizers have been aimed at increasing, on the one hand, the light harvesting capacity of the dyes and, on the other hand, improving their long-term stability under the operation of the device. The first task can be accomplished by increasing the molar extinction coefficients and/or extending the optical absorption up to the near infrared part of the spectrum. The use of thiocyanate ligands typically red-shifts the optical absorption of the dyes. The design of ancillary ligands with extended π-conjugation has the same effect and in addition helps to increase the values of ε. For what concerns the operational stability of the dyes, it has been found that the introduction of long peripheral hydrophobic tails has the fundamental function of repelling the residual water, thus preventing dye desorption form TiO_2_; in addition to the increase in long-term stability, the hydrophobic tails provide an efficient insulation layer that protect TiO_2_ layer from electrolyte, thus reducing the dark current and increasing overall efficiency. The optimization of Ru-based photosensitizers led to DSSC devices with efficiency exceeding 10% and up to 11.5% (Sauvage et al., 2011).

Porphyrin-based photosensitizers were the second class reviewed here and were the subject of deep interest due to their impressive absorption coefficients. The introduction of donor and acceptor moieties to the periphery of the macrocycle core was one of the most important results in the design of high performance porphyrin dyes (Chang et al., 2011): in this way a *push-pull* structure is realized that promotes, on photoexcitation, a directional electron transfer from the donor to the acceptor which is bonded to the semiconductor oxide surface; moreover, the molecular HOMO is in this way far from the anchorage point of the dye on TiO_2_, thus reducing the undesired recombination process between oxidized dye and semiconductor. A very important paper on the development of porphyrins and in general of DSSC dyes concerned the use of a cobalt-based electrolyte to replace the classical iodine-based: the use of a Co(II)/Co(III) redox couple, characterized by a lower value of the redox potential compared to I^−^/I${}_{3}^{-}$, allowed higher values of V_OC_ (approaching or exceeding 1 V) resulting in an increase of the overall efficiency (Yella et al., 2011). Since then, cobalt-based electrolytes are typically used with the aim of maximizing the efficiency of the dye under investigation. The porphyrin-based dyes in combination with cobalt-based redox mediators have afforded remarkable efficiencies: in particular, by using auxiliary electron acceptor to broaden the optical absorption and cover the region between the Soret and the Q bands, outstanding efficiencies of up to 13% were achieved (Mathew et al., 2014). It is worth noticing that cobalt-based electrolytes did not provide satisfactory performance when used in combination with traditional Ru-based sensitizers containing thiocyanate ligands and the reasons were well established (Omata et al., 2015): the design of SCN-free cyclometalated ruthenium dyes is considered the most promising strategy to exploit the favorable redox properties of cobalt complexes mediators (Aghazada et al., 2016).

Metal-free organic dyes represent the third class of photosensitizers reviewed here. For several reasons, in recent years the focus of the DSSC research has shifted to this type of dyes: the simplest and well established synthetic procedures allow a fine-tuning of the optical properties and are more compatible with a low-cost, large-scale production as compared to Ru and porphyrin-based dyes. The high ε values associated with these dyes are highly desirable for applications in solid-state DSSC with long-term operational stability. The development of metal-free dyes has undoubtedly benefited from the previous optimization of the design rules for the other class of DSSC sensitizers: the classical molecular structure of this class of dyes is based on a *push-pull* structure in which an electron donor is bonded through a π-bridge to an electron acceptor (and the whole structure typically covalently decorated by hydrophobic tails). A systematic investigation of these three components has been carried out: TPA and its derivatives are by far the most studied electron donors (Mahmood, 2016) and with indoline type donors provide the most efficient dyes. Several conjugated bridges have been studied, containing auxiliary acceptor groups or rigidified aromatic units to increase planarity (Chaurasia et al., 2015a). The most commonly used electron acceptor group is the cyanoacrylic group which coincides with the anchoring point of the dye on TiO_2_. Very recently a novel trimethoxysilyl anchoring group was proposed by Kakiage group with excellent results: an outstanding efficiency of 14.3% was obtained by this group in a co-sensitized device (Kakiage et al., 2015) and this value represents the current efficiency record for organic based DSSC.

DSSC are expected to enter the market mainly for their superior indoor performance (Freitag et al., 2017) which allows a new range of new applications in the field of portable electronics endowed with autonomous operation. However, an increase in efficiency and stability and a reduction of the overall cost of fabrication is still needed to allow the effective commercialization of this technology. As described, an impressive number of molecular structures has been studied in the last 20 years as potential DSSC photosensitizers and this huge amount of work has produced significant advances in the field of DSSC. Is there room to improve the performance of DSSC photosensitizers? What are the challenges to cope with to make dyes with even better performance? Regarding the stability problems, although proper design rules have been established to realize photosensitizers which favor long-term operational stability, photodegradation of the dyes can still be considered an issue. This issue is shared with other research field employing conjugated dyes in optical or electronic devices; new concepts are recently emerging describing the self-healing of chromophores properties in polymeric media, concepts that could conveniently be implemented in DSSC debate (Ramini and Kuzyk, 2012). If we look at what reported in the literature, it is interesting to notice that, although in the vast majority of the cases the cyanoacrylic moiety has been used as an anchoring group, the organic dye used in the DSSC device with the best performance achieved so far actually bears a trimethoxysilyl anchoring group (Kakiage et al., 2015). The authors claimed that the excellent performance of that dye could be attributed to the superior adsorption properties on TiO_2_ (Kakiage et al., 2014). These works have suggested that the potential of organic dyes bearing silyl anchoring groups as the photosensitizers for DSSCs; in this context a detailed molecular design of different silyl-anchor groups is still necessary and could be considered a fertile ground to be explored on the road to even more performing dyes.

Another interesting point that emerges from the analysis of Tables 1–4 is that, with few exceptions (Yum et al., 2013; Ren et al., 2018), most of the best performing dyes have absorption maxima in the 450–550 nm range, so they are orange/red dyes. One of the future task toward the implementation of DSSC devices with improved efficiency is the design of novel and stable dyes capable of significantly absorbing at longer wavelengths (up to NIR) and injecting electrons into the conduction band of TiO_2_ with a high yield: such an achievement could allow the realization of co-sensitized devices with a real panchromatic absorption and an obvious positive influence on the overall efficiency. An intrinsic problem in making efficient photosensitizers that absorb over 600 nm is that this type of dye is typically characterized by an extremely stable LUMO orbital, such that the electron injection into the conduction band of TiO_2_ is unfavoured (Maglione et al., 2016b). A different approach can be used to overcome this problem, consisting in the realization of a p-type DSSC device in which the role of the phosensitizers is to inject a hole from its HOMO orbital into the valence band of the p-type semiconductor (typically, NiO), so that the LUMO stability is no longer a problem. In recent years, a growing number of papers on p-type DSSC has been published (Odobel and Pellegrin, 2013; Bonomo and Dini, 2016; Wood et al., 2016; Favereau et al., 2017). The interest for this type of device is eventually the fabrication of a tandem DSSC based on the connection of a p-type photocathode with a n-type photoanode. With this configuration it could be possible to harvest a higher fraction of solar photons, by sensitizing the two photoelectrodes with dyes having complementary absorptions. Moreover, the potential of the redox shuttle would not influence the total photovoltage generated by the cell, which is related only to the two photoelectrodes. It has been predicted that a t-DSSC could, in principle, afford an efficiency up to 40% (Xu et al., 2015). A balanced photoanode and photocathode performance is required to obtain high efficient t-DSSCs while, so far, the reported efficiencies of p-DSSCs are significantly lower than the n-type counterparts and reach a maximum PCE of 2.5% (Perera et al., 2015). This technology has been much less investigated than its n-type counterpart and therefore there is still room for its development and the achievement of higher efficiencies.

It must be remembered that DSSC is a multicomponent device and, therefore, not only the photosensitizers but all the components of a DSSC devices need to be optimized to achieve the desired performance. Furthermore, the optimization is rather on the relationship between the different parts than on the single components. The electrolyte is a fundamental part of a DSSC as described in section 2 and has a dramatic influence on the final V_oc_ of the device. The development of new redox shuttles able to reduce the overpotential losses during the dye regeneration is currently under investigation and new systems based on copper (I/II) have been proposed with excellent results (Freitag et al., 2016). At the same time it is clear how solid-state DSSC could offer the better performance in terms of stability; the implementation of new concepts in hole transporting materials based on organic semiconductors could open the way to the development of high performance solid-state devices with increased stability (Freitag et al., 2016). Regarding the cost of the devices, Pt is one of the most expensive materials used in the manufacture of the device and therefore the development of alternative reduction catalysts (as graphene) is currently a very active field of research (Lu et al., 2016a).

In conclusion, relevant progresses in the field of DSSC have been observed in the last 20 years; at the same time the scientific community is still very active in the research and study of new materials and alternative device architectures with the aim of obtaining superior properties in terms of efficiency, stability and costs, so that DSSC technology can spread widely in the market of photovoltaics.

## Author contributions

All authors listed have made a substantial, direct and intellectual contribution to the work, and approved it for publication.

### Conflict of interest statement

The authors declare that the research was conducted in the absence of any commercial or financial relationships that could be construed as a potential conflict of interest.
